# *FIS1* encodes a GA2-oxidase that regulates fruit firmness in tomato

**DOI:** 10.1038/s41467-020-19705-w

**Published:** 2020-11-17

**Authors:** Ren Li, Shuai Sun, Haijing Wang, Ketao Wang, Hong Yu, Zhen Zhou, Peiyong Xin, Jinfang Chu, Tongmin Zhao, Huanzhong Wang, Jiayang Li, Xia Cui

**Affiliations:** 1 Key Laboratory of Biology and Genetic Improvement of Horticultural Crops of the Ministry of Agriculture, Institute of Vegetables and Flowers, Chinese Academy of Agricultural Sciences, Beijing, 100081 China; 2 Sino-Dutch Joint Laboratory of Horticultural Genomics, Institute of Vegetables and Flowers, Chinese Academy of Agricultural Sciences, Beijing, 100081 China; 3 State Key Laboratory of Subtropical Forest Cultivation, Zhejiang Agriculture and Forestry University, Hangzhou, 311300 China; 4 State Key Laboratory of Plant Genomics and National Center for Plant Gene Research (Beijing), Institute of Genetics and Developmental Biology, Chinese Academy of Sciences, Beijing, 100101 China; 5 Vegetable Research Institute, Jiangsu Academy of Agricultural Science, Nanjing, 210014 China; 6 Department of Plant Science and Landscape Architecture, University of Connecticut, Storrs, CT 06269 USA

**Keywords:** Agricultural genetics, Plant breeding

## Abstract

Fruit firmness is a target trait in tomato breeding because it facilitates transportation and storage. However, it is also a complex trait and uncovering the molecular genetic mechanisms controlling fruit firmness has proven challenging. Here, we report the map-based cloning and functional characterization of *qFIRM SKIN 1* (*qFIS1*), a major quantitative trait locus that partially determines the difference in compression resistance between cultivated and wild tomato accessions. *FIS1* encodes a GA2-oxidase, and its mutation leads to increased bioactive gibberellin content, enhanced cutin and wax biosynthesis, and increased fruit firmness and shelf life. Importantly, FIS1 has no unfavorable effect on fruit weight or taste, making it an ideal target for breeders. Our study demonstrates that FIS1 mediates gibberellin catabolism and regulates fruit firmness, and it offers a potential strategy for tomato breeders to produce firmer fruit.

## Abstract (web summary)

Fruit firmness is an important target for breeders and a key determinant of shelf life for many fruits. Here the authors show that mutating tomato FIS1, a GA2-oxidase, increases the concentration of bioactive gibberellins, enhances cutin and wax biosynthesis and increases fruit firmness.

## Introduction

Tomato (*Solanum lycopersicum*) is one of the most important vegetable crops worldwide and has a net production value of over $55 billion^1^. Squashed and softened fruits are the most common defects and have tremendous associated costs in terms of transportation and storage^2^. The *ripening inhibitor* (*rin*) mutation has been introduced into hybrids to produce firm fruits with a long shelf life and improved transportability, but these fruits often have poor taste and are slow to turn color^3,4^. Therefore, enhanced fruit firmness that does not compromise other fruit qualities is a key target characteristic in modern tomato breeding.

Fruit firmness is a complex trait involving numerous physical properties, including cell-wall structure, cellular turgor, and cuticle characteristics^5–8^. Disintegration and degradation of the cell wall are intimately involved in fruit softening. These events are accompanied by the increased expression of genes encoding cell-wall degrading enzymes, including polysaccharide hydrolases, transglycosylases, and other modification proteins, which influence the cell-wall components, resulting in a change in fruit firmness^2^. In addition, the thickness of the cuticle coordinated with expansion of the fruit surface is gradually increased to maintain fruit epidermal structural integrity during fruit growth^9,10^. These dynamic changes in cuticle architecture and composition were proven to be integral elements for fruit firmness alteration^7,9^. Fruit cuticles as composite structures are composed mainly of polymer cutin and cuticular wax^11^. Reducing the contents of these components results in a thinner cuticle, which leads to a decrease in fruit water retention, shelf life, and fruit firmness^9,10,12^. Therefore, the intricate mechanisms underlying fruit firmness need to be disentangled and characterized to effectively manipulate the trait in breeding.

In this regard, several quantitative trait loci (QTLs) associated with firmness have been identified^13–15^. To date, only one locus has been mapped to a 8.6 Mb region on tomato chromosome 2 using introgression lines (ILs), and three pectin methylesterases were nominated as candidate genes^15^. However, precisely identifying the genes associated with fruit firmness remains a daunting task. In recent years, the roles of some genes in fruit firmness have been certified by transgenic tomatoes. Silencing the *pectate lyase* (*PL*) gene in tomatoes enhances fruit firmness and prolongs shelf life^5^. Overexpression or silencing of the *TAGL1* gene in tomato influences cuticle thickness, which is coordinated with the alteration of fruit firmness^16–18^. Although these studies provided usable genes for improving fruit firmness, the limited knowledge of the genes and mechanisms related to fruit firmness still impedes trait selection in breeding.

Gibberellins (GAs) are crucial for a wide range of developmental processes in higher plants. In tomato, GAs likely regulate tomato fruit growth. The application of exogenous GA_3_ to unpollinated ovaries induced fruit set^19^. Transgenic approaches have also proven that changes in GAs levels in tomatoes can induce fruit parthenocarpic development and affect fruit ripening^20–22^. However, as an important hormone, the effect of GAs on other developmental processes of tomato fruit is still unknown. We identified a quantitative trait locus, *qFIRM SKIN 1* (*qFIS1*), that determines fruit firmness between wild and cultivated tomatoes. *FIS1* encodes a GA2-oxidase, and its mutation resulted in an increase in bioactive gibberellin contents in fruits that caused an increase in fruit firmness due to changes in cuticle composition and deposition in the tomato pericarp. Exogenous gibberellin treatment rescued the compression resistance of NIL-*FIS1*^CC^ fruits accompanied by an increase in cuticle thickness. The presence of the *fis1* allele was selected during tomato domestication and contributed to the higher fruit firmness of modern cultivars. Knockout of *FIS1* could increase fruit firmness in tomato. Therefore, the discovery of *FIS1* provides new insight into the mechanism of gibberellin for fruit firmness, making this gene an ideal target for improving fruit firmness in many crops.

## Results

### *qFIS1* is a major locus contributing to fruit firmness in tomato

**Fig. 1:**
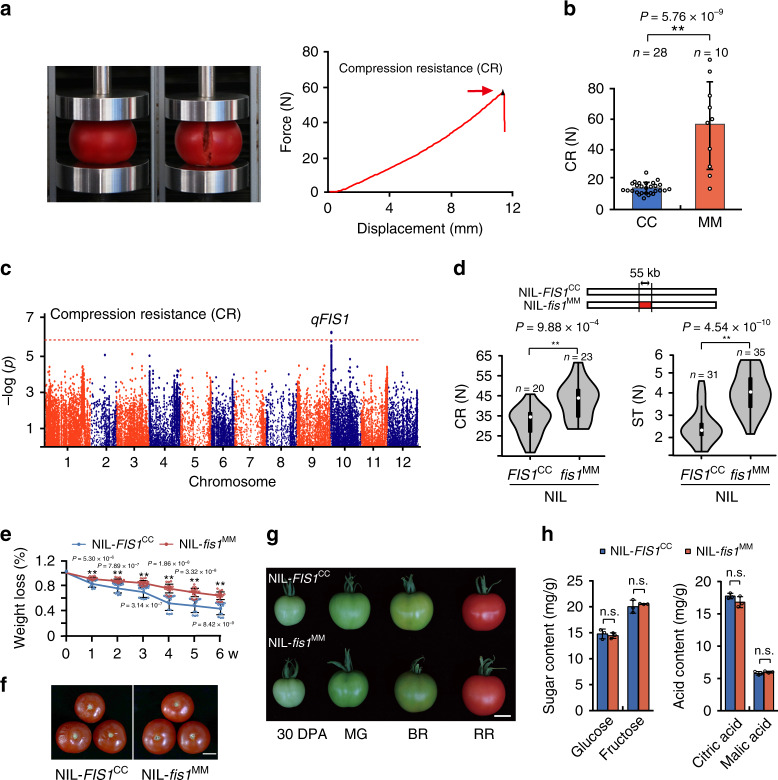
Identification and characterization of *qFIS1*. **a** Photos show the setup for measuring the compression resistance (CR) of tomato red ripe fruits by a texture analyzer. The red arrow indicates the pressure when the fruit is broken. **b** CR of two parental lines. Error bars, mean ± SD. *n* = fruit number. The asterisks indicate a statistically significant difference (two-tailed Student’s *t*-test, **P < 0.01). **c** Manhattan plots of SNPs associated with CR in the RIL population. The horizontal red line indicates the genome-wide significance threshold (1 × 10^−6^), and the *x* axis shows the chromosomal position. **d** Diagram of the DNA region that differed in the two NILs and violin plots of compression resistance (CR) and skin toughness (ST) for NIL-*fis1*^MM^ and NIL-*FIS1*^CC^ red ripe fruits. *n* = fruit number. The asterisks indicate a statistically significant difference (two-tailed Student’s *t*-test, **P < 0.01). **e** Water loss of NIL-*fis1*^*MM*^ and NIL-*FIS1*^*CC*^ red ripe fruits during storage. w, week; Error bars, mean ± SD. Seventeen fruits for NIL-*FIS1*^CC^ and 18 fruits for NIL-*fis1*^MM^ were used for this experiment. The asterisks indicate a statistically significant difference (two-tailed Student’s *t*-test, **P < 0.01). **f** Pictures of NIL-*fis1*^*MM*^ and NIL**-***FIS1*^*CC*^ red ripe fruits stored at room temperature for 2 weeks. Bar =2 cm. **g** NIL-*fis1*^MM^ and NIL-*FIS1*^CC^ fruits at different developmental stages. 30 DPA, 30 days post anthesis; MG, mature green; BR, breaker; RR, red ripe. Bars = 2 cm. **h** Contents of sugars and acids of NIL-*fis1*^MM^ and NIL-*FIS1*^CC^ red ripe fruits. Error bars, mean ± SD. *n* = three biological replicates. n.s., no significant difference (two-tailed Student’s *t*-test, *P* > 0.05).

As a complex trait, fruit firmness is very difficult to quantify, which inhibits the identification of loci. We used a texture analyzer to evaluate compression resistance (CR), which is defined as the pressure when a compressed fruit is broken, reflecting the fruit resistance to squeezing force (Fig. 1a)^23^. We compared the CR of two tomato accessions, *Solanum lycopersicum var. cerasiforme* LA1310 (CC) and *S. lycopersicum* Moneymaker (MM), and found that MM had significantly higher resistance to squeezing (Fig. 1b). We then systematically measured the CR of a stable recombinant inbred line (RIL) population, including more than 200 independent lines derived from these two parents^24^. The distribution of CR resembled an approximately normal distribution (Supplementary Fig. 1a), and two replications showed a high correlation (Supplementary Fig. 1b). An associated study using the whole-genome sequencing data of the RILs identified one significant signal, named *qFIS1* (QTL of *FIRM SKIN*), on the short arm of chromosome 10, which explained 19% of the fruit firmness variation in the RIL population (Fig. 1c).

To further investigate the contribution of *qFIS1*, we crossed two independent recombinant inbred lines, which displayed different phenotypes of CR and shared 90% sequence identity other than the *qFIS1* locus, to construct near-isogenic lines (NILs) by backcrossing for three generations and self-pollinating several times (Supplementary Fig. 1c). The NILs, NIL-*FIS1*^CC^ and NIL-*fis1*^MM^, which were homozygous for the CC or MM *qFIS1* alleles, differed at a 55 kb DNA segment around the locus (Fig. 1d and Supplementary Fig. 1c). We compared CR and skin toughness (ST) between NIL-*FIS1*^CC^ and NIL-*fis1*^MM^ and found that the fruit of NIL-*fis1*^MM^ could endure higher pressure before bursting and puncturing (Fig. 1d), indicating that *qFIS1* contributes to fruit firmness. In line with the fruit firmness, sustained water loss of the NIL-*FIS1*^CC^ fruits was ~2-fold greater than that of NIL-*fis1*^MM^, and the fruit of NIL-*fis1*^MM^ retained good integrity after two weeks of storage (Fig. 1e, f). In addition, the NIL-*fis1*^MM^ fruits showed fewer small stained spots than NIL-*FIS1*^CC^ by toluidine blue staining (Supplementary Fig. 1d). These results indicated that mutation of *FIS1* results in long shelf life in tomato. Notably, fruit ripening was not influence by *qFIS1* (Fig. 1g), and the fruit taste (based on the contents of sugars and acids) also showed no changes in NIL-*fis1*^MM^ (Fig. 1h). Meanwhile, there was no significant difference in any of the following phenotypes between NIL-*FIS1*^CC^ and NIL-*fis1*^MM^: fruit weight, fruit shape, and fruit color (Fig. 1g and Supplementary Fig. 1e). Compared with NIL-*FIS1*^CC^, no alteration of the cell morphology and cell layers of the pericarp that might affect fruit firmness was observed in NIL-*fis1*^MM^ (Supplementary Fig. 1f, g). Therefore, these results indicate that *qFIS1* specifically controls tomato fruit firmness without negative consequences for other fruit traits and ripening.

### *FIS1* controls fruit firmness in tomato

**Fig. 2:**
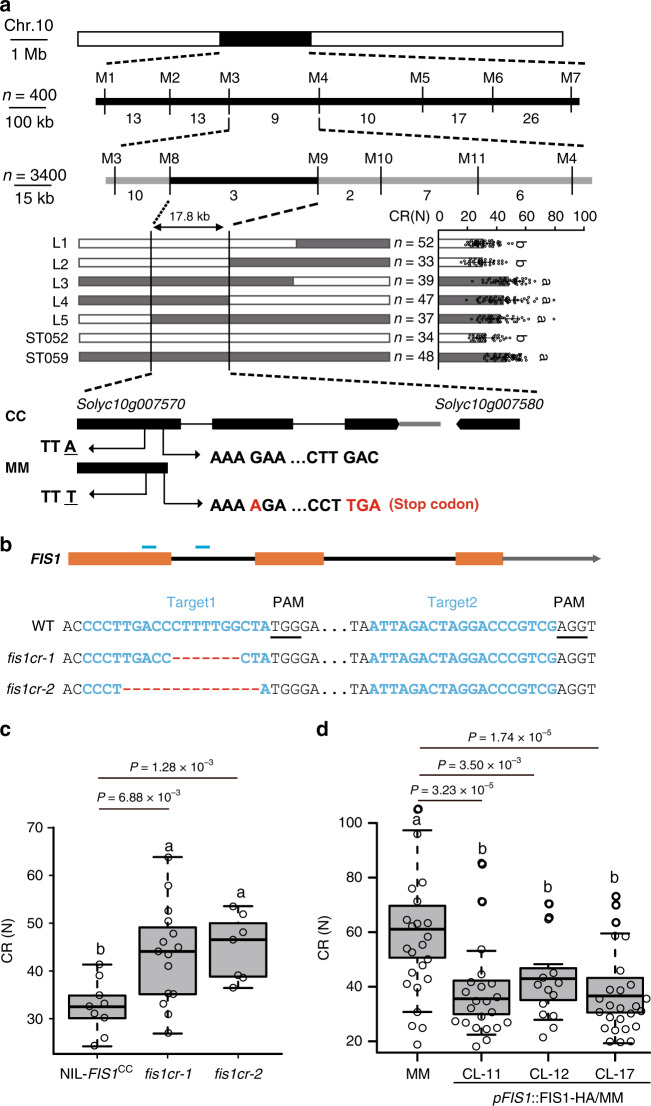
*FIS1* is responsible for fruit firmness formation in tomato. **a** Fine mapping of *qFIS1*. Top panel: positional cloning narrowed *qFIS1* to the DNA segment between markers M8 and M9. The numbers below the bars indicate the number of recombinants. *n* = plant number. Middle panel: high-resolution mapping of *qFIS1* (left) and CR of recombinant progeny (right). Error bars, mean ± SD. *n* = fruit number. Different letters indicate significant differences according to the Tukey–Kramer test (*P* < 0.05). *P* = 3.58 × 10^−18^ (L1 and ST059), *P* = 9.91 × 10^−15^ (L2 and ST059), *P* = 0.55 (L3 and ST059), *P* = 0.28 (L4 and ST059), *P* = 0.07 (L5 and ST059), *P* = 1.65 × 10^−12^ (ST052 and ST059). Bottom panel, genomic diagram showing the two candidate genes. In addition to one SNP, a single nucleotide insertion in the 1st exon of *Solyc10g007570* in MM causes early termination. **b** *FIS1* mutations generated by CRISPR/Cas9 using two single-guide RNAs. Blue lines indicate the target sites of the guide RNAs. Black lines indicate the protospacer-adjacent motif (PAM). The sequences of the two *fis1* mutants in NIL-*FIS1*^CC^ are shown. **c** Box plots of red ripe fruit CR for *fis1cr-1/2* mutants and NIL-*FIS1*^CC^. *n* = 9, 15, and 17 fruits. Box edges represent the 0.25 and 0.75 quantiles, and the bold lines indicate median values. Whiskers indicate 1.5 times the interquartile range. Different letters indicate significant differences according to the Tukey–Kramer test (*P* < 0.05). **d** Box plots of red ripe fruit CR for MM and three transgenic complementation lines (CL-11, CL-12, CL-17). *n* = 23, 22, 13, and 27 fruits. Box edges represent the 0.25 and 0.75 quantiles and the bold lines indicate median values. Whiskers indicate 1.5 times the interquartile range. Different letters indicate significant differences according to the Tukey–Kramer test (*P* < 0.05).

To fine-map *qFIS1*, we conducted high-resolution linkage analysis of 400 individuals of the BC_2_F_3_ population and 3400 individuals of the BC_3_F_3_ population. The *qFIS1* was finally narrowed to a 17.8 kb region between markers M8 and M9 (Fig. 2a and Supplementary Table 1), in which two annotated genes, *Solyc10g007570* and *Solyc10g007580* (Fig. 2a), and a partial promoter region of *Solyc10g007550* were located. Unlike the annotated coding sequence of *Solyc10g007570*, we proved that *Solyc10g007570* has a long first exon including two exons and the first intron annotated in the tomato reference genome (ITAG3.0) (Supplementary Fig. 2a, b). According to the correct sequence, a single nucleotide insertion in the first exon of *Solyc10g007570* in MM led to early termination of translation (Fig. 2a and Supplementary Fig. 2c). In addition, an SNP at the *Solyc10g007580* coding region and fifteen SNPs at the *Solyc10g007550* promoter region were identified when comparing MM and CC genomic sequences. However, the expression levels of *Solyc10g007550* and *Solyc10g007570* in fruits were not different between NIL-*FIS1*^CC^ and NIL-*fis1*^MM^, while *Solyc10g007580* was not expressed in fruits (Supplementary Fig. 2d). These results suggested that *Solyc10g007570* is a candidate gene for the *qFIS1* locus.

To determine the function of *Solyc10g007570*, we mutated *Solyc10g007570* in NIL-*FIS1*^CC^ by CRISPR/Cas9-mediated genome editing. Two null *Solyc10g007570* alleles that showed no off-target mutagenesis on other homologous genes (Supplementary Table 2), named *fis1cr-1* and -*2* with a 7 bp deletion and a 14 bp deletion, respectively, were obtained (Fig. 2b). The fruits of *fis1cr* mutants exhibited higher CR than NIL-*FIS1*^CC^ fruits (Fig. 2c). To further verify whether the variation in the coding sequence of *Solyc10g007570* was responsible for fruit firmness, we constructed a complementation vector containing the coding sequence (CDS) of CC driven by its native promoter and transformed it into the parental line, MM. Compared with the wild-type MM, the CR and ST were obviously reduced in the transgenic lines (Fig. 2d and Supplementary Fig. 2e, f). These results demonstrate that *Solyc10g007570* is *FIS1* and that disruption of *FIS1* function could enhance tomato fruit firmness.

### *FIS1* encodes a GA2-oxidase controlling GA catabolism to regulate fruit firmness

*FIS1* encodes a GA2-oxidase, a gibberellin deactivating enzyme that catalyzes active GAs into inactive products^25^. In Arabidopsis, the specific-tissue expression patterns of GA2-oxidases help modulate bioactive GAs responsible for many aspects of plant development^26^. According to the phylogenetic tree, FIS1 is the sixth member of the GA2-oxidase family and belongs to subgroup I (Supplementary Fig. 3a)^27^. It was highly expressed in tomato fruits from 0 days post anthesis (DPA) to the orange fruit stage, but its transcription was very low in other tissues (Supplementary Fig. 3b). We further analyzed its expression pattern by in situ hybridization and found that *FIS1* was mainly expressed in the placenta, locular tissue, parenchyma cells, and seeds of 10 DPA fruits (Supplementary Fig. 3c). These results suggested that FIS1 may play specific roles in regulating fruit development. Actually, except for fruit firmness, NIL-*FIS1*^CC^ and NIL-*fis1*^MM^ showed no effects on fruit and ripening characteristics, as we previously observed (Fig. 1g and Supplementary Fig. 1e-g), and no change in plant architecture (Supplementary Fig. 4a). Similarly, compared with MM, fruit weight, color, ripening time, and plant height were not obviously altered in the complementation lines (Supplementary Fig. 4b-d), indicating that FIS1 plays a specific function in controlling fruit firmness in tomato.

**Fig. 3:**
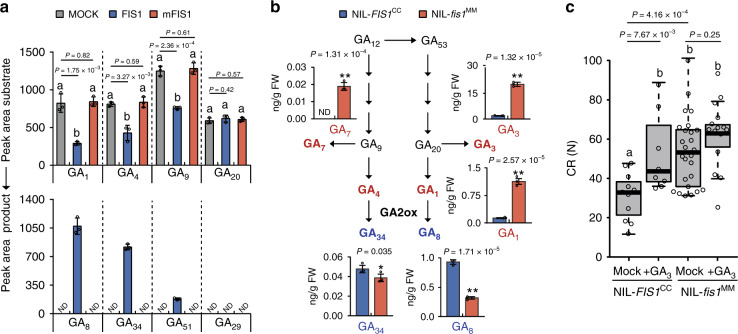
FIS1 regulates fruit firmness via the GA catabolism mediated pathway. **a** Enzymatic activities of recombinant FIS1 and mFIS1 proteins. mFIS1, mutated FIS1. Error bars, mean ± SD. *n* = three biological replicates. Different letters represent significant differences according to the Tukey–Kramer test (*P* < 0.05). **b** The endogenous levels of gibberellins in NIL-*fis1*^MM^ and NIL-*FIS1*^CC^ red ripe fruit pericarp. Error bars, mean ± SD. *n* = three biological replicates. The asterisks indicate a statistically significant difference (two-tailed Student’s *t*-test, *P < 0.05, **P < 0.01). **c** CR of GA-treated or untreated NIL**-***fis1*^MM^ and NIL-*FIS1*^CC^ red ripe fruit*s*. GA was applied at the mature green stage. *n* = 11, 8, 26, and 15 fruits. Box edges represent the 0.25 and 0.75 quantiles, and bold lines indicate median values. Whiskers indicate 1.5 times the interquartile range. Different letters represent significant differences according to the Tukey–Kramer test (*P* < 0.05).

GA2-oxidase catalyzes the hydroxylation of the C-2 of active C19-GAs, which includes GA_1_ and GA_4_ and their immediate precursors GA_20_ and GA_9_, to produce biologically inactive GAs^25^. To characterize whether FIS1 is a functional GA2-oxidase, we detected its enzyme activity using several C_19_ and C_20_ GA substrates. The FIS1 protein was able to convert GA_1_, GA_4_, and GA_9_ to their corresponding 2β-hydroxylated products GA_8_, GA_34_, and GA_51_, respectively, but the mutated FIS1 (mFIS1) was not, indicating that FIS1 is an active enzyme (Fig. 3a). To determine whether the mutation in *FIS1* affects endogenous GAs, we measured GA content in the pericarps of red ripe (RR) fruits of NIL-*fis1*^MM^ and NIL-*FIS1*^CC^. Bioactive GAs, including GA_1_, GA_3_, and GA_7_, were dramatically increased in NIL-*fis1*^MM^ fruits, whereas the metabolic products of GA2-oxidases, including GA_8_ and GA_34_, were decreased in NIL-*fis1*^MM^ relative to NIL-*FIS1*^CC^ (Fig. 3b). These differences indicate that disrupted FIS1 activity reduces gibberellin catabolism in the pericarp of tomato fruit. To test whether exogenous GA could enhance the fruit firmness of NIL-*FIS1*^CC^, we sprayed exogenous GA_3_ on NIL-*FIS1*^CC^ mature green fruits and determined the CR of treated and untreated RR fruits. The CR of treated NIL-*FIS1*^CC^ fruits significantly increased compared to the untreated control. Interestingly, exogenous GA treatment slightly increased but did not significantly influence the CR of NIL-*fis1*^MM^ fruits (Fig. 3c). Taken together, these results demonstrate that FIS1 functions in GA catabolism to regulate fruit firmness without compromising other plant and fruit characteristics.

### FIS1 regulates fruit firmness by influencing cuticle accumulation and deposition

**Fig. 4:**
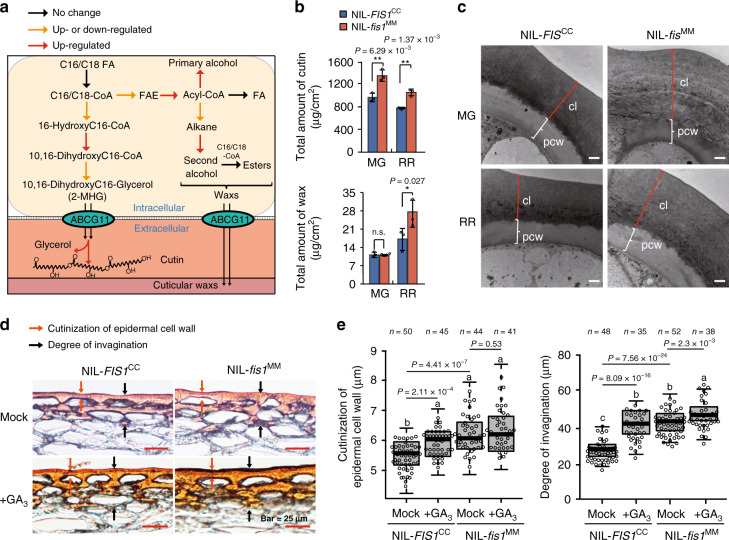
*FIS1* affects cuticle composition and deposition in tomato fruit pericarp. **a** Diagram of the cuticle biosynthesis pathway. Transcriptome data were obtained for NIL-*fis1*^MM^ and NIL-*FIS1*^CC^ 30 DPA and breaker fruits, and the different colored arrows indicate changes in the expression level of known or putative cuticle-modifying genes that function at the indicated biosynthetic steps. The red arrows indicate that all DEGs related to this step were upregulated in NIL-*fis*1^MM^. Orange arrows indicate that some DEGs related to this step were upregulated in NIL-*fis1*^MM^. The black arrows indicate that the transcript levels of structural genes did not differ between NIL-*fis1*^MM^ and NIL-*FIS1*^CC^. **b** Total cutin and wax content in the fruit pericarps of NIL-*fis1*^MM^ and NIL-*FIS1*^CC^. The asterisks indicate a statistically significant difference. Error bars, mean ± SD. *n* = three biological replicates. The asterisks indicate a statistically significant difference (two-tailed Student’s *t*-test, *P < 0.05, **P < 0.01). n.s., no significant difference (two-tailed Student’s *t*-test, *P* > 0.05). MG, mature green; RR, red ripe. **c** Transmission electron microscopy images of NIL-*fis1*^MM^ and NIL-*FIS1*^CC^ fruit pericarp sections. Bar = 2 μm. MG, mature green; RR, red ripe. CL, cuticle layer; PCW, polysaccharide cell wall. Three independent experiments were performed. **d** Cuticle sections stained with Sudan IV to visualize the cutinization of epidermal cell walls and the degree of invagination with or without gibberellin treatment in NIL-*fis1*^MM^ and NIL-*FIS1*^CC^. Bars = 25 µm. Six independent experiments were performed. **e** Quantification of the cutinization of epidermal cell walls and the degree of invagination at the red ripe fruit after GA treatment in NIL-*fis1*^MM^ and NIL-*FIS1*^CC^. *n* = section number. Box edges represent the 0.25 and 0.75 quantiles, and the bold lines indicate median values. Whiskers indicate 1.5 times the interquartile range. Different letters indicate significant differences according to the Tukey–Kramer test (*P* < 0.05).

To investigate how FIS1 regulates firmness, we performed RNA-seq using the fruit pericarps of the NILs at 30 DPA and breaker (BR) fruit. A principal component analysis (PCA) showed that the NIL-*fis1*^MM^ and NIL-*FIS1*^CC^ samples were located closely in 30 DPA fruits, while they departed in BR fruits (Supplementary Fig. 5a). A total of 938 genes at the 30 DPA stage and 1818 genes at the BR stage showed more than 1.5-fold expression differences between MM and CC NIL alleles (Supplementary Fig. 5b and Supplementary Data 1). Gene ontology (GO) analysis showed that genes involved in metabolic processes and gibberellic acid-mediated signaling pathways were significantly enriched among up- or downregulated genes, respectively (Supplementary Fig. 5c, d). Moreover, genes in the cutin, suberine, and wax biosynthesis pathways were upregulated based on Kyoto Encyclopedia of Genes and Genomes (KEGG) analysis (Supplementary Fig. 5e). Therefore, considerable numbers of genes encoding catalytic enzymes at different steps in cutin and cuticular wax biosynthetic pathways were upregulated in NIL-*fis1*^MM^ at 30 DPA or BR fruits compared to NIL-*FIS1*^CC^ (Fig. 4a). For example, the cytochrome P450 genes, *CYP86A69* (*Solyc08g081220*) and *CYP77A-LIKE* (*Solyc03g119200*) participate in cutin biosynthesis^28,29^, *GDSLs* (*Solyc03g111550* and *Solyc04g081770*) are probably required for extracellular polymerization and cutin deposition^30,31^, and *CERs* (*Solyc07g006680, Solyc01g088400* and *Solyc11g067190*) required for cuticular wax biosynthesis^11^. The expression levels of these genes were further analyzed by reverse-transcription quantitative real-time PCR (Q-PCR) in the pericarps of two NILs. Obvious increases in their transcripts were observed in NIL-*fis1*^MM^ consistent with RNA-seq (Supplementary Fig. 6 and Supplementary Data 1). These results suggest that FIS1 might control cuticle biosynthesis of fruit to influence fruit firmness^9^.

To test whether the biosynthesis of cutin and cuticular wax in the pericarp was affected by the variation in *FIS1*, we measured the cuticle components in NIL-*fis1*^MM^ and NIL-*FIS1*^CC^. The contents of total cutin (Fig. 4b) and some monomers, including 10,16-DiOH hexadecanoic (2-MHG), which is the main monomer, were significantly increased by ~30% in both mature green and red ripe fruits of NIL-*fis1*^MM^ compared to that of NIL-*FIS1*^CC^ (Supplementary Table 3). In addition, the total wax amount was also higher in NIL-*fis1*^MM^ than in NIL-*FIS1*^CC^ RR fruits, and two main classes of wax, alcohols, and alkanes, showed nearly 82% and 67% increases in NIL-*fis1*^MM^, respectively (Fig. 4b and Supplementary Table 3). Moreover, we investigated the thickness of the cuticle including the cutinization of epidermal cell walls and the degree of invagination that affects the cuticle properties by histological staining^32^. The characteristics of cutinization of the epidermal cell wall and the degree of invagination clearly revealed cuticle thickening in mature green and red ripe fruits of NIL-*fis1*^MM^ (Supplementary Fig. 7a, b). Consistent with this, a dramatic increase in cuticle thickness in NIL-*fis1*^MM^ was also observed by transmission electron microscopy (TEM). However, no obvious difference in the ultrastructure of the epidermal cell wall was observed (Fig. 4c and Supplementary Fig. 7c). As we previously observed, GA treatment could enhance fruit firmness in NIL-*FIS1*^CC^. We also measured the contents of cutin and wax and analyzed the cuticle morphology of NIL-*FIS1*^CC^ after GA treatment. The total cutin content of RR fruits was significantly enhanced in NIL-*FIS1*^CC^ but not in NIL-*fis1*^MM^ after GA treatment compared with untreated fruits (Supplementary Fig. 7d). In contrast, the wax amount was increased in GA-treated fruits of both NIL-*FIS1*^CC^ and NIL-*fis1*^MM^ compared to the untreated fruits (Supplementary Fig. 7d). In accordance with the increases in cutin and wax contents, epidermal cell cutinization and epidermal invagination were also significantly thickened in NIL-*FIS1*^CC^ after GA treatment (Fig. 4d, e). When we complemented the MM with *FIS1*, the cuticle thickness of transgenic RR fruits became thinner than that of MM fruits (Supplementary Fig. 7e, f). Collectively, these data indicate that FIS1 regulates fruit firmness by influencing cuticle accumulation and deposition in tomato fruits.

### Selection of *FIS1* enhances tomato fruit firmness during domestication

**Fig. 5:**
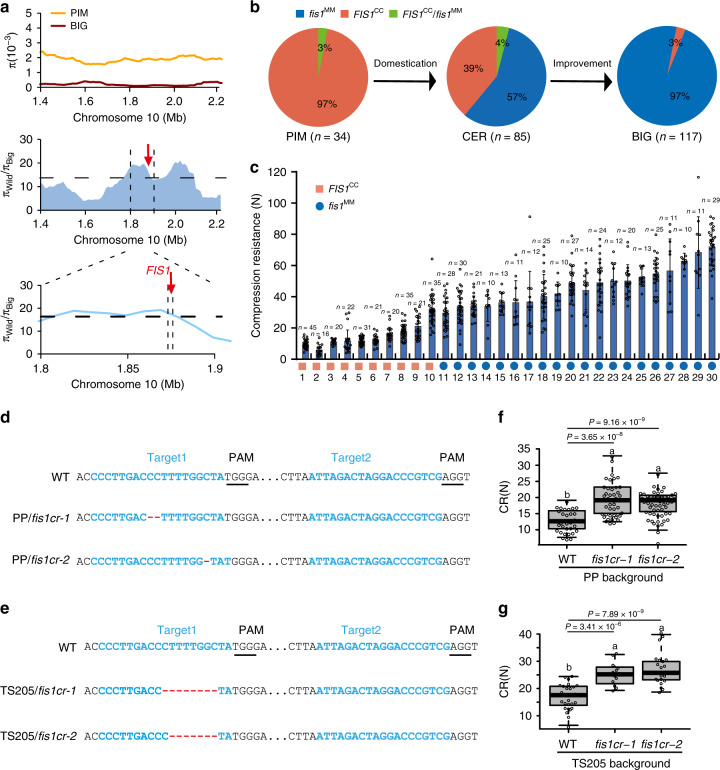
*FIS1* is selected during tomato domestication and is a target for the improvement of fruit firmness. **a** Scan for selection signature surrounding the *FIS1* gene. Top: nucleotide diversity (π) of wild species (orange line) and big-fruit cultivars (red line). Middle: π ratio of the wild species to big-fruit cultivars (blue region) for a region on chromosome 10. The horizontal dashed lines indicate the genome-wide top 5% ratio cutoff for candidate domestication sweeps. The vertical black dashed lines (bottom) delimit *FIS1*. **b** Frequency distribution of the *FIS1* genotype in natural populations including 236 resequenced tomato accessions. PIM: *Solanum pimpinellifolium*, CER: *S. lycopersicum var. cerasiforme*, BIG: Big-fruit tomatoes. **c** CR of 20 cultivated tomatoes with the *fis1*^MM^ allele and 10 cherry tomatoes with the *FIS1*^CC^ allele. Error bars, mean ± SD. *n* = fruit number. **d** *FIS1* mutations generated by CRISPR-Cas9 using two single-guide RNAs in PP. Blue lines indicate the target sites of the guide RNAs. Black lines indicate the protospacer-adjacent motif (PAM). The sequences of PP/*fis1cr-1/2* mutants are shown. **e** *FIS1* mutations generated by CRISPR/Cas9 using two single-guide RNAs in TS205. The sequences of TS205/*fis1cr-1/2* mutants are shown. **f** Box plots of red ripe fruit CR for PP/*fis1cr-1/2* mutants and PP. *n* = 31, 42, and 58 fruits. Box edges represent the 0.25 and 0.75 quantiles, and the bold lines indicate median values. Whiskers indicate 1.5 times the interquartile range. Different letters indicate significant differences according to the Tukey–Kramer test (*P* < 0.05). **g** Box plots of red ripe fruit CR for TS205/*fis1cr-1/2* mutants and TS205. *n* = 28, 12, and 23 fruits. Box edges represent the 0.25 and 0.75 quantiles and the bold lines indicate median values. Whiskers indicate 1.5 times the interquartile range. Different letters indicate significant differences according to the Tukey–Kramer test (*P* < 0.05).

Fruit firmness is a key target for cultivar selection in tomato breeding. Analysis of *FIS1* variation in 166 tomato varieties and 53 *S. pimpinellifolium* accessions^33^ indicated that the *FIS1* locus was under selection during tomato domestication (Fig. 5a). Moreover, *FIS1* is located in a previously reported selective sweep^33^. Approximately 97% of the cultivated tomatoes and 57% of the early domesticated cherry tomatoes were homozygous for *fis1*^MM^ alleles, whereas all of the analyzed wild species were homozygous for *FIS1*^CC^ except the heterozygous alleles (Fig. 5b), indicating that the *FIS1* allele was positively selected during tomato domestication. To determine the contribution of *FIS1* in domesticated tomatoes, we quantified the effect of allelic variation at *FIS1* on tomato fruit firmness in 30 tomato accessions collected from different regions worldwide (Supplementary Table 4). We found that tomato accessions with the *fis1*^MM^ genotype had higher CR than tomato species with the *FIS1*^CC^ genotype (Fig. 5c). Thus, *FIS1* is a target selected in tomato breeding for fruit firmness.

### Genome editing of *FIS1* improves tomato fruit firmness

Disruption of FIS1 function could increase tomato fruit firmness with negligible effects on other fruit traits. Therefore, *FIS1* could be a very useful target to improve fruit firmness in tomato breeding. To validate this hypothesis, we mutated *FIS1* in two tomato accessions by CRISPR/Cas9-mediated genome editing. One of them belongs to wild *S. pimpinellifolium* named PP, and the other is a cherry tomato cultivar, TS205^33^. Two independent *fis1cr* mutants were obtained in the PP or TS205 background, which were named PP/*fis1cr-1* and PP/*fis1cr-2* with 2 and 1 bp deletions (Fig. 5d), respectively, and TS205/*fis1cr-1* and TS205/*fis1cr-2* with 8 and 7 bp deletions, respectively (Fig. 5e). As expected, all of these *fis1cr* mutants exhibited higher CR than their respective controls (Fig. 5f, g). With the increase in fruit firmness, the cuticles of RR fruits were significantly thickened in these *fis1cr* mutants compared with PP or TS205 (Supplementary Fig. 8a-c). At the same time, the fruit weights showed no obvious difference (Supplementary Fig. 8d, e). These results confirm that *FIS1* is an ideal target for the improvement of fruit firmness in tomato.

## Discussion

During the process of tomato domestication, not only did fruit yield improve significantly but also fruit texture changed greatly compared with its progenitors. Our present findings demonstrate the following causal sequence of changes related to selection at the *FIS1* locus during tomato domestication: disrupted FIS1 function due to a 1-bp frameshift insertion, increased accumulation of bioactive GAs in the fruit pericarp, enhanced cutin biosynthesis and cuticle accumulation, and increased tomato fruit firmness with negligible effects on other phenotypes (Supplementary Fig. 8f).

Fruit firmness is an important trait that affects consumer acceptance, fruit transportability, and shelf life. Nevertheless, fruit firmness is a highly complex trait that is determined by a number of factors involving numerous pathways. Extensive studies on fruit firmness have focused on the remodeling and degradation of the cell wall^5,8^. In addition, cuticle properties have also been suggested to contribute to fruit firmness. The fruit cuticle is a continuous hydrophobic structure composed predominantly of two components: cutin and wax^11^. Dynamic changes in cutin and wax compositions during fruit development are associated with cuticle accumulation that could affect fruit firmness in a direct way by acting as a supporting barrier under tension, or indirectly regulating fruit water status^7,9^. The mutated and knocked-down genes involved in cuticle biosynthesis often result in a thinner cuticle layer, leading to a decrease in fruit firmness^7,9,30^. In our study, the cuticle thickness (Supplementary Fig. 7a, b) and cutin and wax contents of the NIL-*fis1*^MM^ fruits (Supplementary Table 3) were strikingly increased compared to those of CC allele fruits that is the cause of the enhanced fruit firmness in MM allele. In contrast, the CR of mature green fruits displayed no significant differences between NIL-*fis1*^MM^ and NIL-*FIS1*^CC^, but an obvious increase in ST could be observed in NIL-*fis1*^MM^ (Supplementary Fig. 9). One possibility is that the mature green fruits are hard and difficult to break. However, the skin toughness reflects the change in the cuticle layer at the mature green stage. Moreover, cuticle as a protective skin is highly correlated with fruit water loss and shelf life^9,10^. Downregulated cutin/wax contents lead to reduced water retention capacity in fruits^10^. Similarly, the increase in cutin/wax contents in NIL-*fis1*^MM^ causes fruits to have a long shelf life (Fig. 1e). Thus, the change in fruit cuticle is the reason for the alteration of fruit firmness caused by the variation in *FIS1*.

Gibberellin is an important hormone that controls fruit development. Changes in GA catabolism in transgenic tomato and the application of exogenous GA could affect fruit ripening^21^. As a GA2ox protein, FIS1 is a critical catabolic enzyme that maintains endogenous GA levels by catalyzing bioactive GA into bioinactive GA^26^. Mutation of this gene in NIL-*fis1*^MM^ causes an increase in bioactive GA and results in an enhancement of fruit firmness in tomato (Fig. 3b) without significant effects on fruit ripening and morphology. Our work reveals GA may play a role in regulating fruit firmness during fruit ripening. However, *FIS1* was mainly expressed in the mesocarp instead of the exocarp during fruit development according to our in situ results (Supplementary Fig. 3c) and tomato laser microdissection RNA-seq data^34^. It is difficult to explain how FIS1 could specifically affect the cuticle layer of tomato fruit exocarp. Actually, GAs can be transported from synthetic tissue into other tissues to perform their functions^35^. One possibility is that more bioactive GAs are transported into exocarp in *fis1* mutants. Moreover, FIS1 and the other five GA2oxs display redundant and specific functions in tomato fruit development. Our results and those from tomato fruit RNA-seq datasets indicate that four *GA2ox* genes are expressed in fruits but with different spatial-temporal expression patterns, especially *Solyc07g056670* (Supplementary Fig. 10). This latter gene is highly expressed during ripening in both the tomato inner and outer epidermis^34^. Therefore, revealing the functions of Solyc07g056670 in GA metabolism and fruit development will help us to address this question.

Bioactive GAs induce the transcription of cutin and wax synthetic genes in many species^36,37^. Our RNA-seq assay also indicated that a dozen genes related to cutin and wax biosynthesis are upregulated with the accumulation of bioactive GA in NIL-*fis1*^MM^ (Supplementary Data 1). These upregulated genes were required for the biosynthesis and extracellular polymerization of cuticle^30^ that determine fruit viscoelastic behavior and fruit firmness^7,9^. Therefore, our results suggest that FIS1 manipulates cuticle biosynthetic processes to regulate fruit firmness. Previous studies have revealed that *SHINEs* (*SHNs*) act in cuticle accumulation by regulating the expression levels of several downstream genes associated with cuticle biosynthesis in *Arabidopsis* and tomato^28,36^. Although we found that *SlSHN2* displayed a higher expression level in NIL-*fis1*^MM^ than in NIL-*FIS1*^CC^, whether *SlSHN2* is one of the transcription factors that participates in the FIS1-mediated cuticle biosynthetic pathway still needs to be explored in the future.

Fruit firmness is always the main target in tomato breeding. Ripening mutants have been used as parents to generate F_1_ hybrids in order to confer firm fruit and long shelf life^3,4^. However, the hybrids also inherited poor traits, such as bad flavor and poor color^4^. Moreover, cell-wall-modifying related genes have been used as targets for obtaining hard fruit. Except for pectate lyase (*PL*)^5^, silencing most of these genes yielded slight or even undetectable effects on fruit firmness^8,38^. Therefore, few genes could be used to improve fruit firmness to date. Using the CRISPR-edited method generates *fis1cr* mutants in tomato accessions that could improve fruit firmness effectively without taking unfavorable changes in fruit quality (Fig. 5d–g and Supplementary Fig. 8d, e). Thus *FIS1* is a useful target to enhance fruit firmness in tomato. Taken together, our work provides new insights into the biological mechanism underlying fruit firmness and raises the prospect of improving fruit hardness.

## Methods

### Plant materials and growth conditions

RILs containing 219 individual lines were derived from *S. lycopersicum* cv. Moneymaker (MM) and *S. lycopersicum* var. *cerasiforme* (CC) were used as materials for association analysis, as we described previously^24^. To clone the gene, we generated a BC_3_F_2_ population derived from the cross between two RILs, ST052 and ST059, which shared 90% sequence identity other than the *qFIS1* locus. Cultivated tomato MM, NIL-*FIS1*^CC^, PP (*S. pimpinellifolium*), and cherry tomato TS205 were used for the transgenic experiments. Field experiments were performed in a greenhouse at the Nankou experimental station in Beijing, China. Seedlings were grown in a commercial nursery for 30–40 days and then transplanted to fields.

### Phenotypic evaluation

CR and ST were measured using an Instron 5542 texture analyzer according to a previously reported method^23^. Eight red ripe fruits of each plant and four plants of each line were used for CR and ST measurements.

More than ten plants of each genotype were used to evaluate fruit weight. The average fruit weight of each plant was represented by the average fruit weight of all fruits on the second inflorescence. The average fruit weight of ten plants represented the fruit weight of each genotype.

For cell layer and cell area measurements, 10–15 fruits of each NIL line were picked at the mature green stage. Five free-hand sections from the equator of each fruit were used to analyze the average cell area by staining with 0.1% toluidine blue for 10 s. Images were taken with a Leica DFC450C microscope. The cell layer was measured in the abaxial–adaxial direction of the pericarp. The average cell area was measured in two (*α* and *β*) regions and was calculated using the following formula: average cell area = area (*α* + *β*)/total cell number (*α* + *β*).

### Content analysis of sugars and acids

More than 8 red ripe fruits were collected from each NIL line for sugar and acid analysis. The mixed fruit pericarp was ground in liquid nitrogen, and then 100 mg of ground powder was diluted in 2 ml water. The following was added to the water-soluble tomato pericarp extracts: saccharide internal standard (1 mg/ml arabinose water) and acid internal standard (0.5 mg/ml lactic acid). After sonication and centrifugation, the samples were filtered through a 0.22 µm polyethersulfone ultrafiltration membrane and mixed with an equal volume of acetonitrile for analysis. Saccharide and acid contents were measured by UPLC-MS/MS (ACQUITY UPLC I-Class-Xevo TQ-S Micro, Waters). For saccharide analysis, an ACQUITY UPLC BEH Amide 1.7 µm column was used as the analytical column (2.1 × 100 mm; Waters). The mobile phase was composed of acetonitrile as solvent A and 1 mg/ml ammonium hydroxide as solvent B. The temperatures of the column and autosampler were 60 °C and 4 °C, respectively. Each saccharide was separated by increasing the solvent B concentration from 10% to 20% over 6 min after the first 2 min of the run at 10% using a flow rate of 0.2 ml/min, followed by washing with 10% solvent B for 2 min. For acid analysis, an ACQUITY UPLC HSS T3 1.8 µm column was used as the analytical column (2.1 × 100 mm; Waters). The mobile phase was composed of acetonitrile containing 1 mg/ml formic acid as solvent A and water containing 1 mg/ml formic acid as solvent B. The temperatures of the column and autosampler were 25 °C and 4 °C, respectively. The acid elution was performed at a flow rate of 0.1 ml/min with 90% B for 5 min. Data analysis was performed using MassLynx V4.1 (Waters).

### Water loss and cuticle permeability measurements

A total of 10–15 fruits of each NIL line were picked at the RR stage and then stored at room temperature for 6 weeks. Fruit weight was measured every week. Water loss was calculated as the percentage of the decrease in fruit weight. For measurements of cuticle permeability, MG fruits of NILs were collected and dropped in 1% toluidine blue solution staining for 12 h as described in Hovav et al.^39^.

### Associated analysis and gene mapping

The genome sequencing data of the RIL population and their parents were obtained from our previously published work^24^. The paired-end reads of MM, CC, and the RILs were mapped to the tomato reference genome (SL2.50). SNP calling was performed on the alignment results using the Genome Analysis Toolkit (GATK) version 3.1.1 and Picard package version 1.119^40^. The calling was performed according to the following steps: (1) unmapped reads were deleted, (2) duplicate reads were deleted, (3) alignment using the IndelRealigner package in GATK was conducted, and then (4) SNP calling for each sample was performed using the UnifiedGenotyper package in GATK, with a minimum base quality score of 20. To ensure the quality of SNP calling in MM and CC, SNPs were filtered further with the VariantFiltration package in GATK using parameters QD < 2.0 || FS > 60.0 || MQ < 40.0 || MQRankSum < −12.5 || ReadPosRankSum < −8.0, and SNPs with DP < 10 were also removed. The remaining SNPs between MM and CC were used as the index for SNP calling in the 219 RILs. The SNPs in the 219 RILs were filtered with the Lowqual tag marked by the GATK UnifiedGenotyper package. To infer the missing genotype at an SNP site of an individual line, 20 SNPs flanking the target SNP in other lines of the population were compared with the individual line. If all of the lines having the same genotype for the flanking SNPs as the individual line had the same genotype at the target SNP, this SNP genotype was inferred/imputed to the individual line. The RIL association study was conducted using the imputed information for the SNPs of the 219 RILs by the compressed mixed linear model implemented in GAPIT^41^. The cutoff *P* value was set as 1E−6. For fine mapping, based on the genotypes of 400 BC_3_F_2_ individuals derived from the cross of ST052 and ST059, two recombinant lines of the RILs and 22 recombinant plants were obtained for the progeny test and the *FIS1* gene was mapped between markers M3 and M4. An additional 28 recombinant plants were screened from 3400 BC_3_F_3_ individuals by genotyping with six markers, including M3, M4, M8, M9, M10, and M11 (Supplementary Table 1). Together with the progeny test results, we delimited *qFIS1* to a 17.8 kb region between the Indel markers M8 and M9. The markers used for mapping are listed in Supplementary Table 1.

### Construction design and plant transformation

For CRISPR-Cas9 constructs, two gRNAs, target1 and target2, were designed using the CRISPR-P v2.0 tool (http://cbi.hzau.edu.cn/CRISPR2/). A pair of primers, P1, containing two sgRNAs and *AarI* recognition sites was used to amplify the Target1_U6-26t_SlU6p_Target2 fragments using the pCBC_DT1T2_SlU6p vector as the template, which was revised from the pCBC_DT1T2 vector^42^ by replacing *AtU6p* with *SlU6p*^43^. Then, the PCR product was digested with *AarI* to generate the gRNA cassette, and inserted into pCAMBIA2300_35 S_Cas9_SlU6p_sgRNA for CRISPR/Cas9 construction, which was modified from pCAMBIA2300 by inserting a SlU6p_sgRNA after the NOS terminator and a rice optimized Cas9^44^ after the 35S promoter. The complementary construct contained a 2420-bp promoter region of the *FIS1* gene and a 1008-bp coding sequence, which were amplified from CC using the primers P4 and P5, respectively. The DNA fragment was inserted into the pCAMBIA2300-HA vector to generate the construct *pFIS1*::FIS1-HA. CRISPR and complementary plasmids were introduced into *Agrobacterium tumefaciens AGL1* competent cells and transformed into NIL-*FIS1*^CC^, PP, TS205, and MM by *Agrobacterium*-mediated transformation. The transgenic lines were confirmed by PCR and sequencing. All experiments were performed using homozygous lines from the T_2_ generation without T-DNA integration. The PCR primers used for plasmid construct and transgenic line determination are listed in Supplementary Table 1. The off-target sites were predicted using the online tool (http://crispr.mit.edu), and then the sequences of targets were amplified using genomic DNA of transgenic plants as template and then analyzed by sequencing.

### Phylogenetic trees and motif recognition

The amino-acid sequences of GA2oxs of Arabidopsis and tomato were downloaded from the NCBI database, and aligned using ClustalW. A phylogenetic reconstruction analysis was performed based on the neighbor-joining method using PHYML version 3.0 under the JTT evolution model. The reliability of the obtained trees was tested by bootstrapping with 500 replicates. Motif analysis was performed using the EMBL-EBI database (http://smart.embl-heidelberg.de/). Multiple-sequence alignments were performed with a gap open penalty of 10 and gap extension penalty of 5 using DNAMAN 6.0 and ClustalW 2.0^45^.

### In situ hybridization

In situ hybridization was performed as described in Scott et al. with modification^46^. Cubes of ~5-mm per side tissue from NIL-*FIS1*^CC^ 10 DPA fruits were fixed for 24 h at 4 °C in freshly prepared 4% (w/v) paraformaldehyde buffered with phosphate-buffered saline (PBS, pH 7.2). Fixed tissues were dehydrated in a graded ethanol:Histochoice (H2779-1L, Sigma) series and impregnated with Paraplast (P3683-1kg, Sigma). Dewaxed thin sections (10 μm) were hybridized with the hydrolysis probes at a concentration of 2 ng/μl in hybridization buffer for 12 h at 55 °C. Hybridization buffer for each slide: 20 μl of 10× buffer (3 M NaCl, 0.1 M Tris-HCl pH 6.8, 0.1 M NaPO_4_, 50 mM EDTA), 40 μl of 50% dextran sulfate (S4030, Merck-Millipore), 80 μl of deionized formamide (0606-100mL, Amresco), 4 μl of 50× Denhardt’s solution (30915-25mL, Sigma), 39 μl of formamide (V900064-500 mL, Sigma), and 2 μl of tRNA (10109541001, Roche) in 200 μl of DEPC-H_2_O. Color development complete sections were observed using a fluorescence microscope (DM5500, Leica).

For the *FIS1* probe, the *FIS1* coding region was amplified with the primers P16: 5′-GCATCTATTTTGGGTCTCAATCCA-3′ and 5′-CATCAGGACATGGAGGATAATG-3′, and cloned into pEAZY-T3 (TransGen), which contained T7 and SP6 promoter sequences. M13F and M13R (N53002, Thermo Fisher) were used to amplify recombinant fragment containing cDNA from the *FIS1* and T7 and SP6 promoters. In vitro transcription was performed with T7 RNA polymerase using the purified PCR product as the template to generate the antisense or sense probe.

### Cuticle staining

Fruits were collected for each NIL line at mature green and red ripe fruit stages. The pericarp equator was cut into 2-mm cubes for fixation in FAA buffer (water:formaldehyde:glacial acetic acid:ethanol 1:2:10:7). The fixed samples were dehydrated in a graded series of ethanol (70, 85, 95, and 100%), followed by a xylene/ethanol series (xylene/ethanol 1:3, 1:1, 3:1, and 100% xylene). Xylene was replaced gradually using a paraffin/xylene series (paraffin/xylene 1:3, 1:1, and 3:1) at 37 °C, 45 °C, and 60 °C for 12 h, respectively. Finally, the sample solution was replaced with fresh paraffin every 12 h for four times. A Leica RM2235 microtome was used to cut 10 µm sections for staining with 0.05% Sudan IV. Cuticle thickness was calculated using ImageJ.

### Transmission electron microscopy (TEM)

For the TEM assay, a total of 6–8 mature green or red ripe fruits of each NIL line were used. Cubes of ~2-mm per side were excised from the fruit equator. Methods of sample fixation, embedding, and slicing were performed as described with modification^12^. Samples were fixed in primary fixative containing 2.5% (v/v) glutaraldehyde in 0.05 M phosphate buffer (pH 6.8) for 4–6 h at room temperature. Samples were washed with phosphate buffer and postfixed in 1% (v/v) osmium tetroxide and 1.5% potassium ferricyanide. Fixed samples were washed with water and then dehydrated through a gradient ethanol series (50, 70, 80, 90, and 100%). Prepared samples were infiltrated with propylene oxide resin mixtures and polymerized in 100% resin for 48 h at 60 °C. Ultrathin sections (80–100 nm) were stained with 2% uranyl acetate and Reynold’s lead citrate and viewed with a H-7500 (Hitachi) transmission electron microscope. Images were analyzed using ImageJ software.

### Content analysis of cutin monomer and wax

Cutin monomer analysis was performed according to a previously described method with modification^9^. Isolated peels (10-cm^2^ area) were incubated at 85 °C in 2-propanol with 0.01% (w/v) 2,6-di-tert-butyl-4-methylphenol for 30 min, and then the solution was replaced with fresh 2-propanol. After 2 h, the cuticles were washed in a chloroform/methanol series (chloroform/methanol 2:1, 1:1, and 1:2), for 12 h at each step. After washing with methanol to remove the chloroform, the cuticles were depolymerized in 8 ml of anhydrous methanol containing 7.5% (v/v) methyl acetate and 4.5% (w/v) sodium methoxide at 60 °C. Methyl heptadecanoate and v-pentadecalactone were added as internal standards. After 2 h, the reaction was cooled and 1 ml of acetic acid was added to adjust pH, followed by two dichloromethane extractions (10 ml) to remove methyl ester monomers. The organic phase was washed three times with 0.9% NaCl (w/v), dried with 2,2-dimethoxypropane, and dried under nitrogen gas. The residue was prepared for GC by derivatization using N,O,-bis(trimethylsilyl)-trifluoroacetamide (BSTFA). The samples were dried and then dissolved in n-hexane:toluene (1:1) prior to analysis on an Agilent GC-MS instrument (7890B-5977A, Agilent) with 30 m × 0.25 mm HP-5 MS columns and helium as the carrier gas. GC was performed with temperature-programmed automatic injection at 60 °C, holding for 5 min at 60 °C, temperature increase to 230 °C at a rate of 2 °C min^−1^, and holding for 40 min at 230 °C.

The cuticular wax analysis was modified from a previously reported method^9^. The chloroform-soluble cuticular wax extracts from tomato pericarp of a known area (100 cm^2^) were evaporated to dryness under a stream of nitrogen gas, and the dried residue was prepared for GC by derivatization using N,O,-bis(trimethylsilyl)-trifluoroacetamide (BSTFA). The experiment was carried out with temperature-programmed automatic injection at 70 °C, held for 5 min at 70 °C, raised by 2 °C min^−1^ to 180 °C, and held for 5 min at 180 °C.

### Enzyme activity of recombinant FIS1 protein

In vitro protein expression was performed according to previously described method with minor modifications^47^. The full-length cDNAs of *FIS1* amplified from MM and CC were cloned into the *pIX-HALO* (ABRC) vector using the primer pair, P17 (Supplementary Table 1). The *pIX-HALO* vector was used as a control. Confirmed constructs were used for recombinant protein expression in wheat germ TNT expression systems (L4140, Promega). For the enzyme activity assay, the procedure was adapted from previous studies with slight modification^48,49^. Fifty or seventy-seven microliters of total protein was incubated with GA metabolites (10 ng of GA_1_, 2 ng of GA_4_, 2 ng of GA_9_, 2 ng of GA_20_, OlChemIm) in 100 μl of reaction buffer containing 100 mM Tris-HCl (pH 7.5), 1 mM FeSO_4_, 10 mM 2-oxoglutarate, 10 mM ascorbate, and 5 mM DTT at 30 °C for 6 h. After incubation, 100 ml of methanol was added and mixed. After centrifugation, the supernatant was collected for analysis by LC-MS/MS (ACQUITY UPLC I-Class-Xevo TQ-S Micro, waters) with an ACQUITY UPLC BEH C18 (2.1 mm × 50 mm, 1.7 μm) column according to a method modified from previous studies^50^. Acetonitrile was mobile phase A and water with 0.05% formic acid was mobile phase B. The run was at a flow rate of 0.2 ml/min with an initial 3% of solvent A. Solvent A changed from 3% to 65% in 17 min, then from 65 to 90% in 1.5 min and was held at 90% for 1 min; it was returned to 3% from 90% in 1.5 min. Finally, solvent A was held at 3% for 1.5 min. The column and injection room (FTN) temperatures were all 20 °C. The injection volume was 1 μl for all samples. Mass was set in negative ion mode for GA analysis. The ESI source parameters were set as follows: electrospray capillary voltage, 3.0 kV; source temperature, 150 °C; desolvation temperature, 600 °C; desolvation gas flow, 650 L/h; cone gas flow, 10 L/h. The mass acquisition was performed in multiple reaction monitoring for 22.5 min.

### Content analysis of endogenous GAs

Pericarps of 10–15 red ripe fruits of the NIL lines were used for GA content measurement, as described in previous work, with minor modifications^51^. Five hundred milligrams of the ground plant powder was extracted with 90% aqueous methanol (MeOH). As internal standards for GA content measurement, 2 ng of each D-labeled GA compound was added to the extracting solvents. The MAX cartridge (Waters Corporation, Milford, USA) was activated and equilibrated with MeOH, water, 5% NH_4_OH, and 90% MeOH, and MCX (Waters Corporation, Milford, USA) was activated with MeOH, water, and 90% MeOH. The crude extracts were loaded onto the tandem cartridges connected with an adapter. The MAX cartridge was disconnected, rinsed with 5% NH_4_OH in 5% MeOH, and then rinsed with MeOH. GA compounds were eluted with 90% MeOH containing 2% FA. The eluent was dried under a nitrogen stream and redissolved in 40% MeOH prior to UPLC-MS/MS analysis. GA analysis was performed on a quadrupole linear ion trap hybrid mass spectrometer (QTRAP 6500, AB) equipped with an electrospray ionization (ESI) source coupled with a UPLC (Waters). The UPLC inlet method and ESI source parameters were set as in a previous report^51^. GAs were detected in negative multiple reaction monitoring (MRM) mode.

### Exogenous GA_3_ treatment

The fruits of NILs were marked on the flower opening day and treated with 2.5 mM GA_3_ (63492-1G, Sigma-Aldrich) by spraying from the MG stage. All fruits were treated every 3 days, for four times total. Fruits were collected at the RR stage for measurement of fruit weight, fruit compression resistance, and cuticle. Water treatment was the control.

### RNA-seq analysis

The RNA-seq reads were aligned to the tomato genome (ITAG2.4) by STAR v2.5.3^52^, and their features were counted by feature Counts v 1.5.3^53^, as described in a previous paper^54^. Using the statistical package DEGseq in R version 3.0.3, the MA-plot-based method was used to calculate *P* values that were adjusted using the Benjamini–Hochberg procedure. The fold change between two libraries was calculated as FPKM (fragments per kilobase of transcript sequence per million base pairs sequenced). The thresholds for the identification of differentially expressed genes (DEGs) were as follows: FPKM > 1 in any tissue, fold change >1.5 or <0.666, and Benjamini–Hochberg adjusted *P* value < 0.05. The primers used for DEG verification are listed in Supplementary Table 1. GO and KEGG analyses of DEGs were performed using their best homologous genes in Arabidopsis with DAVID (The Database for Annotation, Visualization, and Integrated Discovery, https://david.ncifcrf.gov/).

### Identification of selective sweeps

To identify the selection region around *FIS1*, the SNPs near the *FIS1* locus (Chr10:1.4–2.2 Mb, SL2.50) in the tomato genome were obtained. We measured the level of nucleotide diversity (π) using a 100-kb window with a step size of 10 kb in wild species (wild) and big-fruit cultivars (big). The ratios of nucleotide diversity between wild and big (πwild/πbig) were also calculated. According to the strategy described in a previous study^33^, the top 5% of ratios were used as the cutoff for sweeps.

### Reporting summary

Further information on research design is available in the Nature Research Reporting Summary linked to this article.

## Supplementary information

Supplementary Information

Descriptions of Additional Supplementary Files

Supplementary Data 1

Reporting Summary

## Data Availability

The RNA sequencing datasets generated in this study and genomic sequencing datasets of RILs have been deposited in the Sequence Read Archive (SRA) under the accession number PRJNA559455 and SRP093370. Other data supporting our findings are available in the manuscript file or from the corresponding author upon request. Source data are provided with this paper.

## Source data

Source Data
