# Supplementary material for: FIS1 encodes a GA2-oxidase that regulates fruit firmness in tomato
Source: Nat Commun. 2020 Nov 17;11:5844. doi: 10.1038/s41467-020-19705-w (PMC7673020; doi:10.1038/s41467-020-19705-w)
Supplement: Supplementary file 1 — Supplementary Information [file 41467_2020_19705_MOESM1_ESM.pdf]

Supplementary Information List

Supplementary Figure 1. *FIS1* is the key gene that regulates fruit firmness.

Supplementary Figure 2. *Solyc10g007570* is a candidate gene for the *qFIS1* locus.

Supplementary Figure 3. *FIS1* encodes GA2ox and is expressed in different fruit developmental stages.

Supplementary Figure 4. Phenotypes of NILs and transgenic complementation plants.

Supplementary Figure 5. Analysis of DEGs between NIL-*fis1*^{MM} and NIL-*FIS1*^{CC} fruits.

Supplementary Figure 6. Expression levels of several known and putative cuticle-modifying genes in 30 DPA and breaker fruits.

Supplementary Figure 7. Cuticle thickness and contents of cutin and wax in NILs and transgenic complementation plants.

Supplementary Figure 8. Mutation of *FIS1* affects cuticle thickness and the model of the FIS1 effect on fruit firmness during fruit domestication.

Supplementary Figure 9. Skin toughness (ST) and compression resistance (CR) of NIL-*fis1*^{MM} and NIL-*FIS1*^{CC} mature green fruits.

Supplementary Figure 10. Expression pattern of *FIS1* homolog genes in CC.

Supplementary Table 1. List of markers and primers used in the work.

Supplementary Table 2. Mutation frequency analysis at predicted off-target sites of *FIS1*.

Supplementary Table 3. The contents of cutin and wax compositions in fruit pericarps of NIL-*fis1*^{MM} and NIL-*FIS1*^{CC}.

Supplementary Table 4. List of tomato accessions used to evaluate compression resistance.

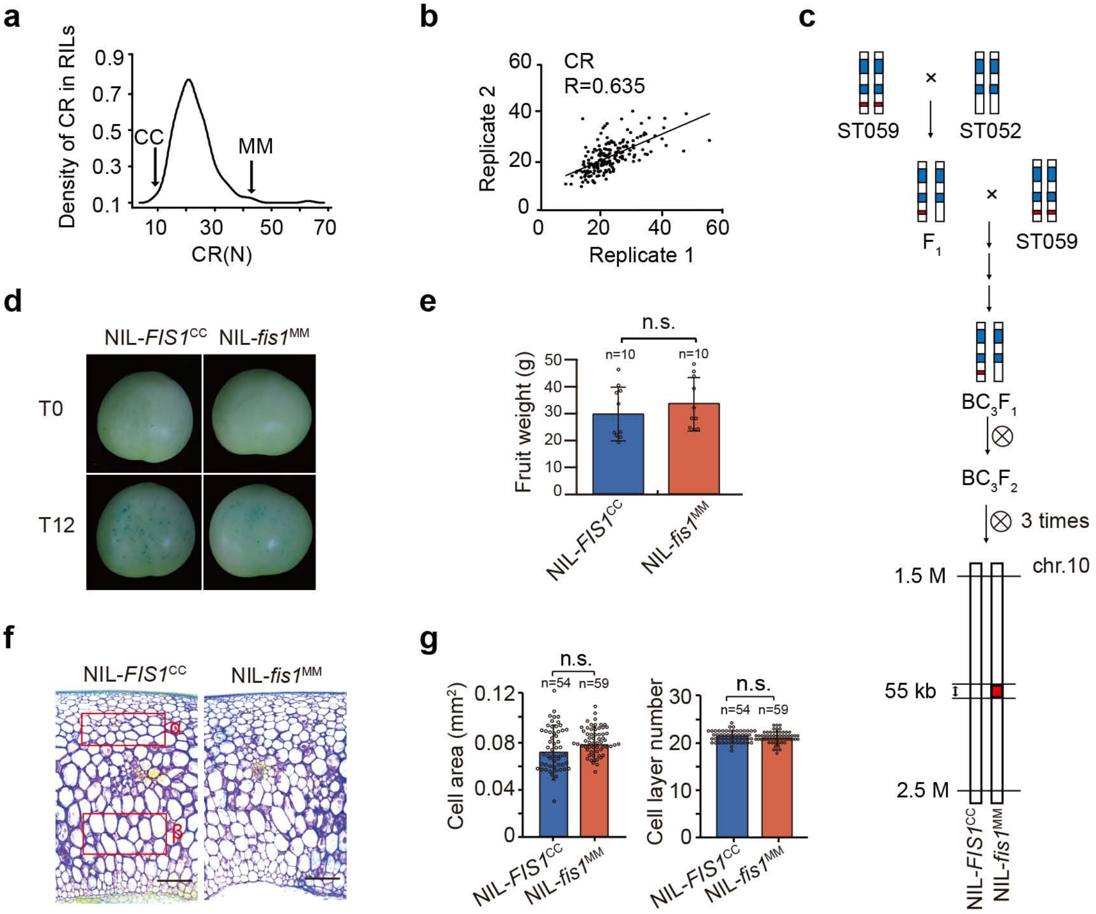

Supplementary Fig 1. *FIS1* is the key gene that regulates fruit firmness. a, Density of CR values in the RILs. The two arrows indicate the CR values of the MM and CC parental lines. **b,** Correlation between two replicates of CR measurement. **c,** Diagram of the production of NILs by crossing two RIL lines. **d,** Uptake of toluidine blue across the cuticle layer of mature green fruit. T, toluidine blue staining time. **e,** Fruit weight of NIL-*fis1*^{MM} and NIL-*FIS1*^{CC}. Error bars, mean ± SD. n = plant number. n.s., no significant difference (two-tailed Student's *t*-test, $P > 0.05$). **f,** Cell morphology and arrangement in pericarps of NIL-*fis1*^{MM} and NIL-*FIS1*^{CC}. Bar = 500 μm. The average cell size was measured of α and β boxes. **g,** Quantification of the cell area and cell layers of NIL-*fis1*^{MM} and NIL-*FIS1*^{CC} cells. Error bars, mean ± SD. n = section number. n.s., no significant difference (two-tailed Student's *t*-test, $P > 0.05$). CR, compression resistance.

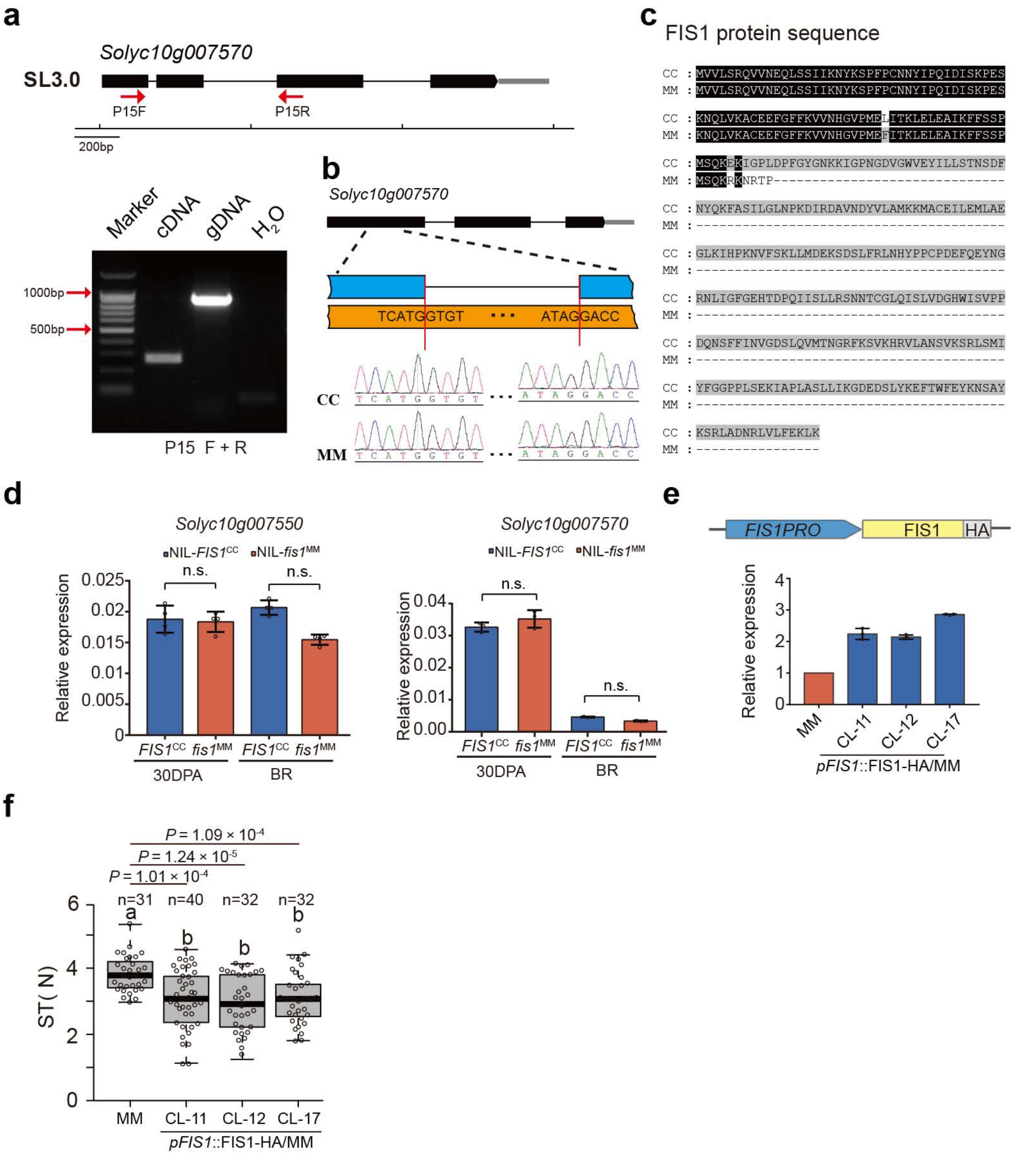

Supplementary Fig 2. *Solyc10g007570* is a candidate gene for the *qFIS1* locus. a, The DNA fragments amplified by primers P15F and P15R using cDNA and genomic DNA of CC. The annotated gene structure of *Solyc10g007570* (SL3.0) is shown. The black blocks and the lines indicate exons and introns, respectively. The gray block indicates the 3'UTR. **b,** Sanger sequencing results of the DNA bands. The gene structure of *Solyc10g007570* is shown. The blue blocks and black line indicate the annotated exons and introns annotated in SL3.0. The orange block indicates the actual exon. The color peaks show the Sanger sequencing signals. **c,** Sequence alignment of FIS1 proteins in CC and MM parental lines. The dashed line indicate the deletion of FIS1 protein in MM. **d**, Relative expression of *Solyc10g007550* and *Solyc10g007570* in 30 DPA and BR fruits. Error bars, mean ± SD, n = three biological replicates. n.s., no significant difference (two-tailed Student's *t*-test, $P > 0.05$). **e,** Relative expression of *FIS1* in MM and three transgenic complementation lines (CL-11, CL-12, CL-17). Error bars, mean ± SD, n = three biological replicates. n.s., no significant difference (two-tailed Student's *t*-test, $P > 0.05$). **f,** Box plots of fruit CR for MM and three transgenic complementation lines (CL-11, CL-12, CL-17). Box edges represent the 0.25 and 0.75 quantiles and bold lines indicate median values. Whiskers indicate 1.5 times the interquartile range. n = fruit number. Different letters indicate significant differences according to the Tukey-Kramer test ($P < 0.05$). DPA, days post anthesis; BR, breaker; CR, compression resistance.

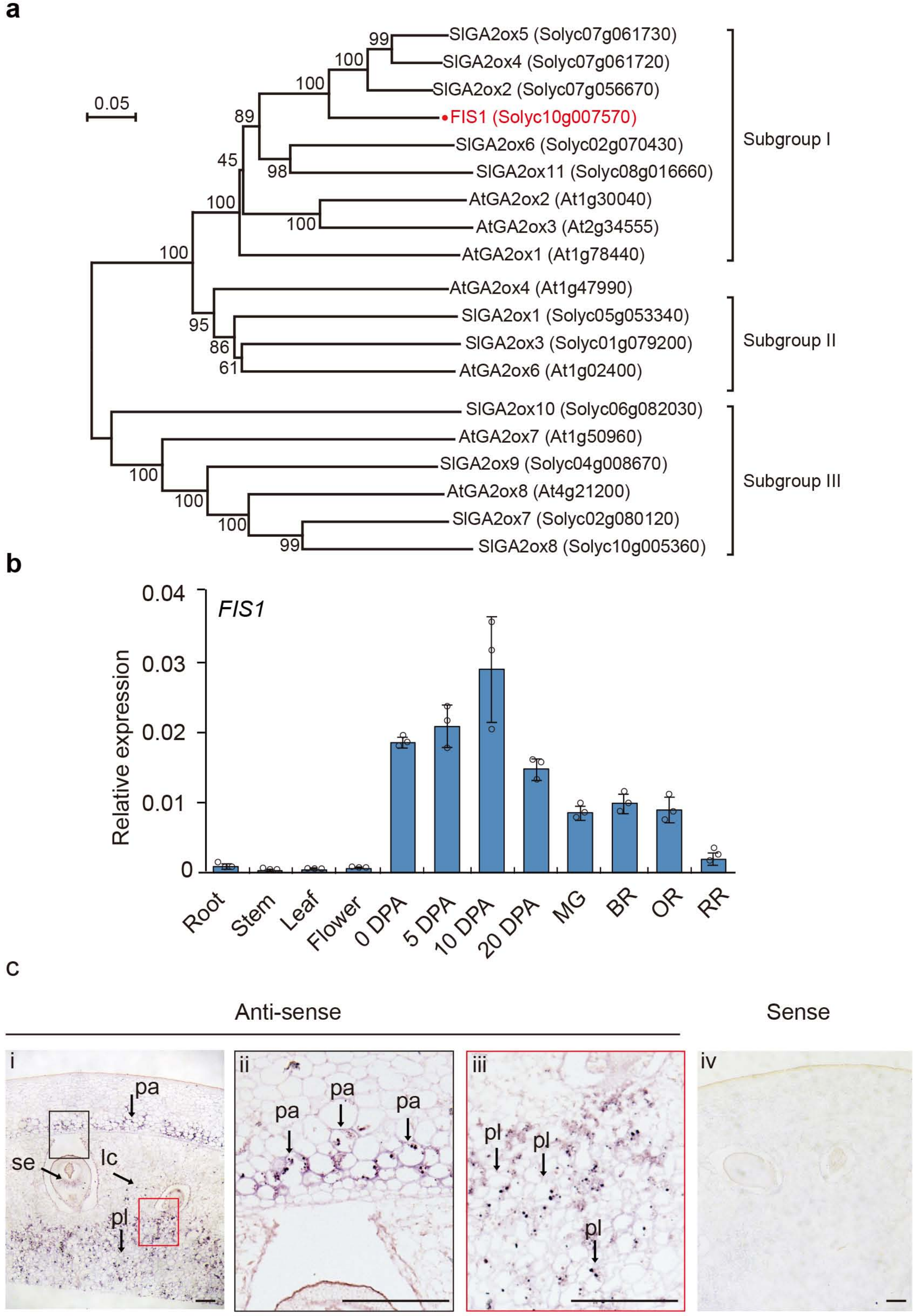

Supplementary Fig 3. ***FIS1*** **encodes GA2ox and is expressed in different fruit developmental stages.**
a, The phylogenetic tree of GA2ox members in tomato and Arabidopsis. **b,** Expression pattern of *FIS1* in CC. Error bars, mean ± SD, n = three biological replicates. **c,** mRNA *in situ* hybridization of *FIS1* expression in 10 DPA fruit. i-iii, anti-sense probe; iv, sense probe. ii and iii, detailed images of black and red boxes in image i, respectively. pa, parenchyma cell; pl, placenta cell; lc, locular tissue; se, seed. Bar = 300 μm. The experiment was repeated two times with similar results. DPA, days post anthesis; MG, mature green; BR, bresker; OR, orange; RR, red ripe.

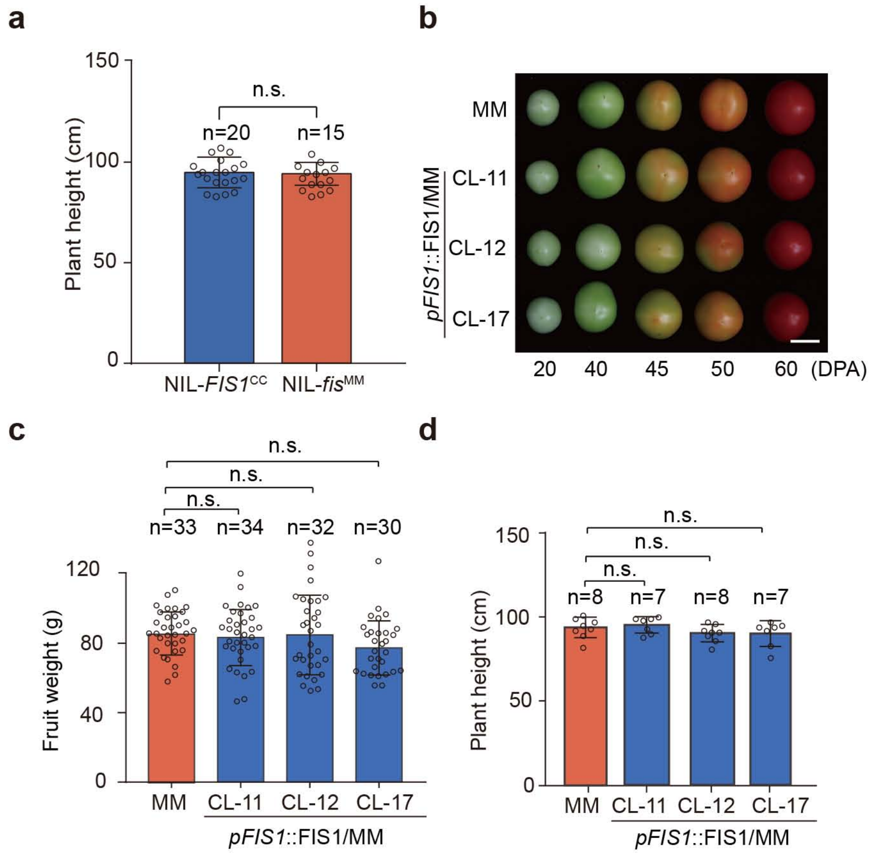

Supplementary Fig 4. Phenotypes of NILs and transgenic complementation plants. a, Plant height of NIL-*fis1*^{MM} and NIL-*FIS1*^{CC}. Error bars, mean ± SD. n = plant number. n.s., no significant difference (two-tailed Student's *t*-test, $P > 0.05$). **b,** Photos of fruits of MM and transgenic complementation lines. Bar = 5 cm. **c,** Fruit weight of MM and transgenic complementation plants. Error bars, mean ± SD. n = plant number. n.s., no significant difference (two-tailed Student's *t*-test, $P > 0.05$). **d,** Plant height of MM and transgenic complementation plants. Error bars, mean ± SD. n = plant number. n.s., no significant difference (two-tailed Student's *t*-test, $P > 0.05$). MM, Moneymaker; CL-11, CL-12, and CL-17, transgenic complementation lines. DPA: day after anthesis.

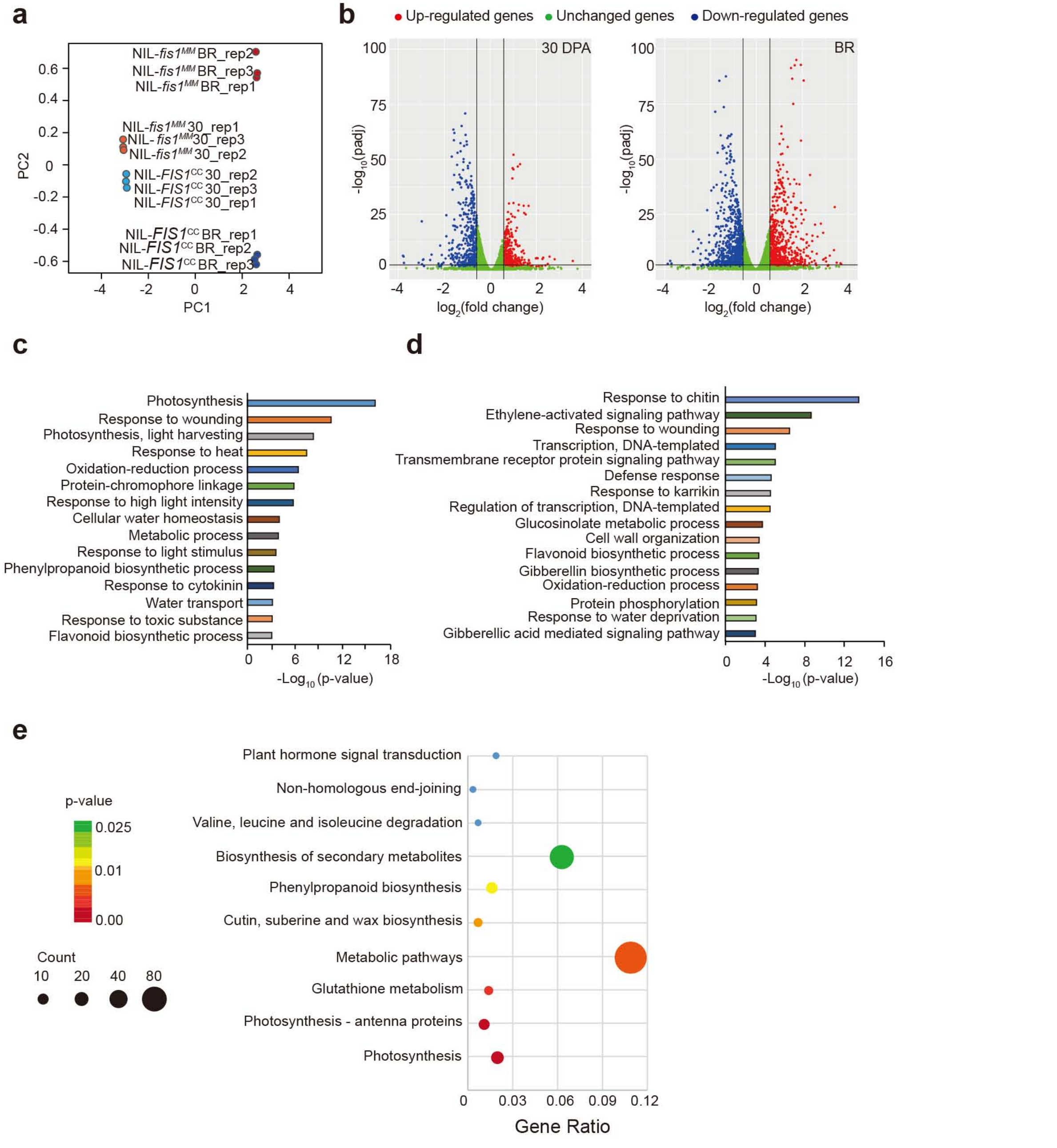

Supplementary Fig 5. Analysis of DEGs between NIL-*fis1*^{MM} and NIL-*FIS1*^{CC} fruits. a, Principal component analysis (PCA) of the RNA-seq datasets. The circles of different colors indicate the samples of 30 DPA and BR fruits of NIL-*fis1*^{MM} or NIL-*FIS1*^{CC}. **b,** Volcano plots were used to visualize the RNA-seq data. Each dot corresponds to a reference gene. Red and blue dots represent upregulated and downregulated genes in NIL-*fis1*^{MM} compared to NIL-*FIS1*^{CC}, respectively. **c and d,** Gene Ontology (GO) terms in the biological processes of upregulated genes **(c)** and downregulated genes **(d)**. *p*-value< 0.001. **e,** Kyoto Encyclopedia of Genes and Genomes (KEGG) analysis of upregulated genes in BR fruits. DPA, day after anthesis; BR, breaker.

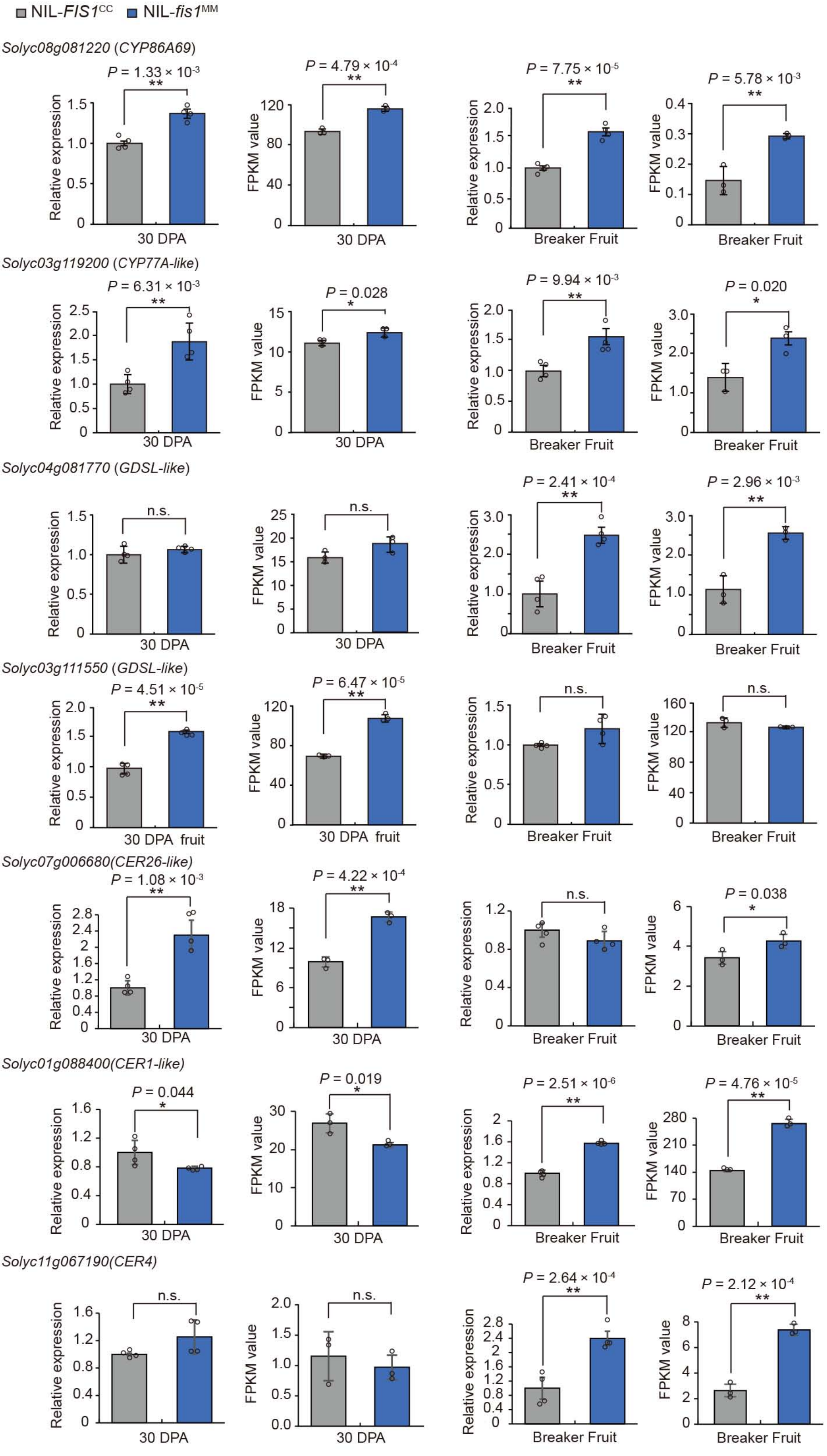

Supplementary Fig 6. Expression levels of several known and putative cuticle-modifying genes in 30 DPA and breaker fruits
Error bars, mean ± SD, Four biological replicates for "relative expression", three biological replicates for "FPKM value". The asterisks indicate a statistically significant difference (two-tailed Student's *t*-test, *, $P < 0.05$, **, $P < 0.01$). n.s., no significant difference (two-tailed Student's *t*-test, $P > 0.05$).

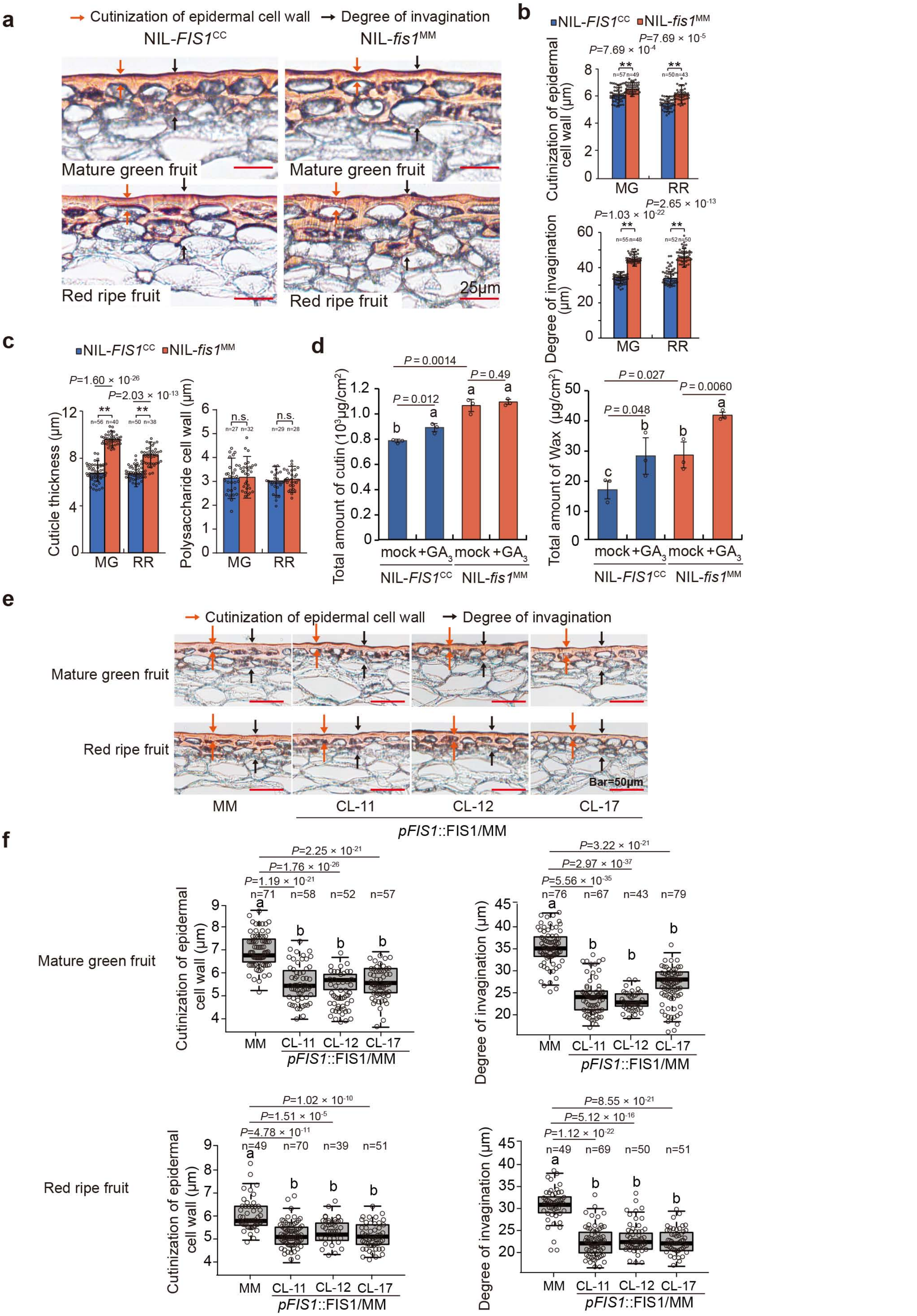

Supplementary Fig 7. Cuticle thickness and contents of cutin and wax in NILs and transgenic complementation plants. **a,** Cuticle sections stained with Sudan IV to visualize the cutinization of epidermal cell walls and the degree of invagination of NIL-*fis1*^{MM} and NIL-*FIS1*^{CC} at MG and RR stages. Bars = 25 μm. The experiment was repeated six times with similar results. **b,** Quantification of epidermal cell wall cutinization and the degree of invagination in NIL-*fis1*^{MM} and NIL-*FIS1*^{CC}. Error bars, mean ± SD. n = section number. The asterisks indicate a statistically significant difference (two-tailed Student's *t*-test, $^{**}P < 0.01$). **c,** Quantification of the thickness of the cuticle and polysaccharide cell wall in NIL-*fis1*^{MM} and NIL-*FIS1*^{CC} MG and RR fruits from transmission electron microscopy images. Error bars, mean ± SD. n = section number. The asterisks indicate a statistically significant difference (two-tailed Student's *t*-test, **, $P < 0.01$). n.s., no significant difference (two-tailed Student's *t*-test, $P > 0.05$). **d,** Content of cutin and wax amount of NIL-*fis1*^{MM} and NIL-*FIS1*^{CC} with or without GA treatment. Error bars, mean ± SD. three biological replicates. Different letters represent significant differences according to the Tukey-Kramer test ($P < 0.05$). **e,** Cuticle sections stained with Sudan IV to visualize the cutinization of epidermal cell walls and the degree of invagination of MM and the three transgenic complementation lines. Bars = 50 μm. The experiment was repeated six times with similar results. **f,** Quantification of epidermal cell wall cutinization and the degree of invagination in MM and three transgenic complementation lines. n = section number. Box edges represent the 0.25 and 0.75 quantiles and bold lines indicate median values. Whiskers indicate 1.5 times the interquartile range. Different letters represent significant differences according to the Tukey-Kramer test ($P < 0.05$). MG, mature green; RR, red ripe.

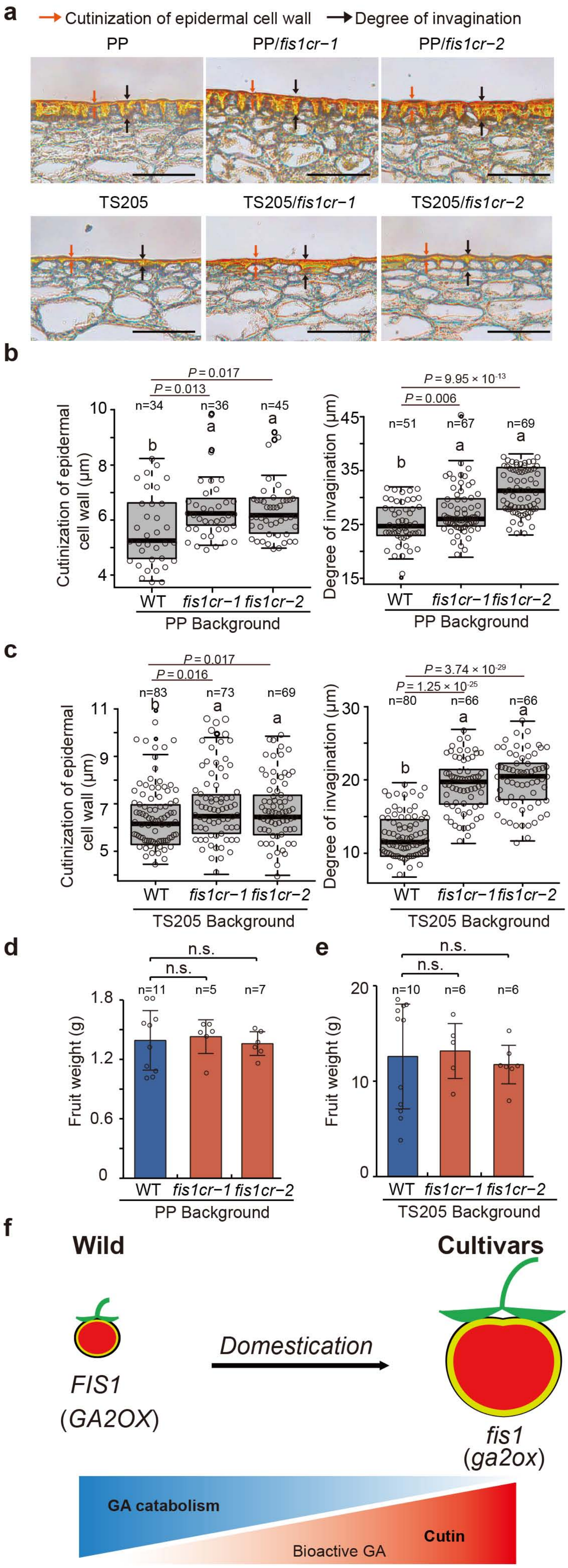

Supplementary Fig 8. Mutation of *FIS1* affects cuticle thickness and the model of the FIS1 effect on fruit firmness during fruit domestication. **a,** Cuticlesections stained with Sudan IV to visualize the cutinization of epidermal cell walls and the degree of invagination of *fis1* alleles and their controls. Bars = 50 μm. **b** and **c,** Quantification of epidermal cell wall cutinization and the degree of invagination in *fis1cr* mutants in the PP (**b**) or TS205 (**c**) background. n = section numbers. Box edges represent the 0.25 and 0.75 quantiles and bold lines indicate median values. Whiskers indicate 1.5 times the interquartile range. Different letters indicate significant differences according to the Tukey-Kramertest ($P < 0.01$). **d** and **e,** Fruit weight of *fis1cr* alleles in PP (**d**) or TS205 (**e**) background. Error bars, mean ± SD. n = plant number. n.s., no significant difference (two-tailed Student's *t*-test, $P > 0.05$). **f,** Proposed model of *FIS1* regulation of tomato fruit firmness during tomato domestication.

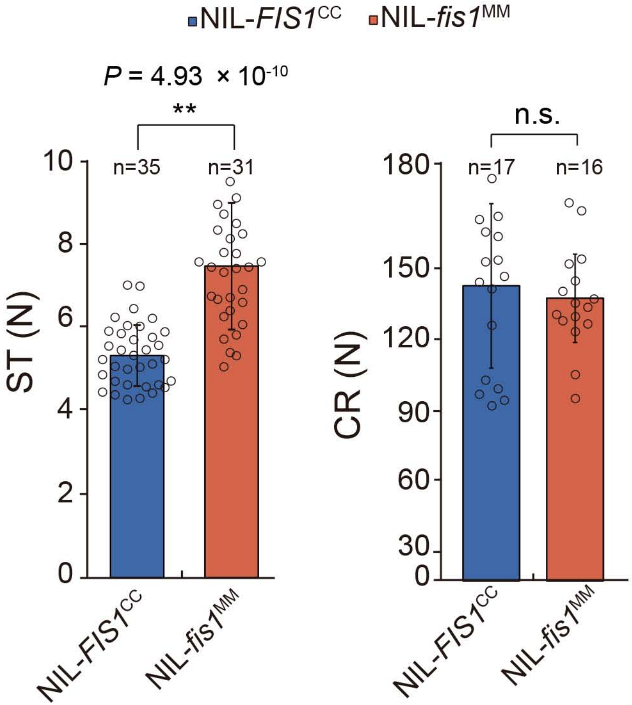

Supplementary Fig 9. Skin toughness (ST) and compression resistance (CR) of NIL-*fis1*^{MM} and NIL-*FIS1*^{cc} mature green fruits. Error bars, mean ± SD. n = fruit number. The asterisks indicate a statistically significant difference (two-tailed Student's *t*-test, **, $P < 0.01$). n.s., no significant difference (two-tailed Student's *t*-test, $P > 0.05$).

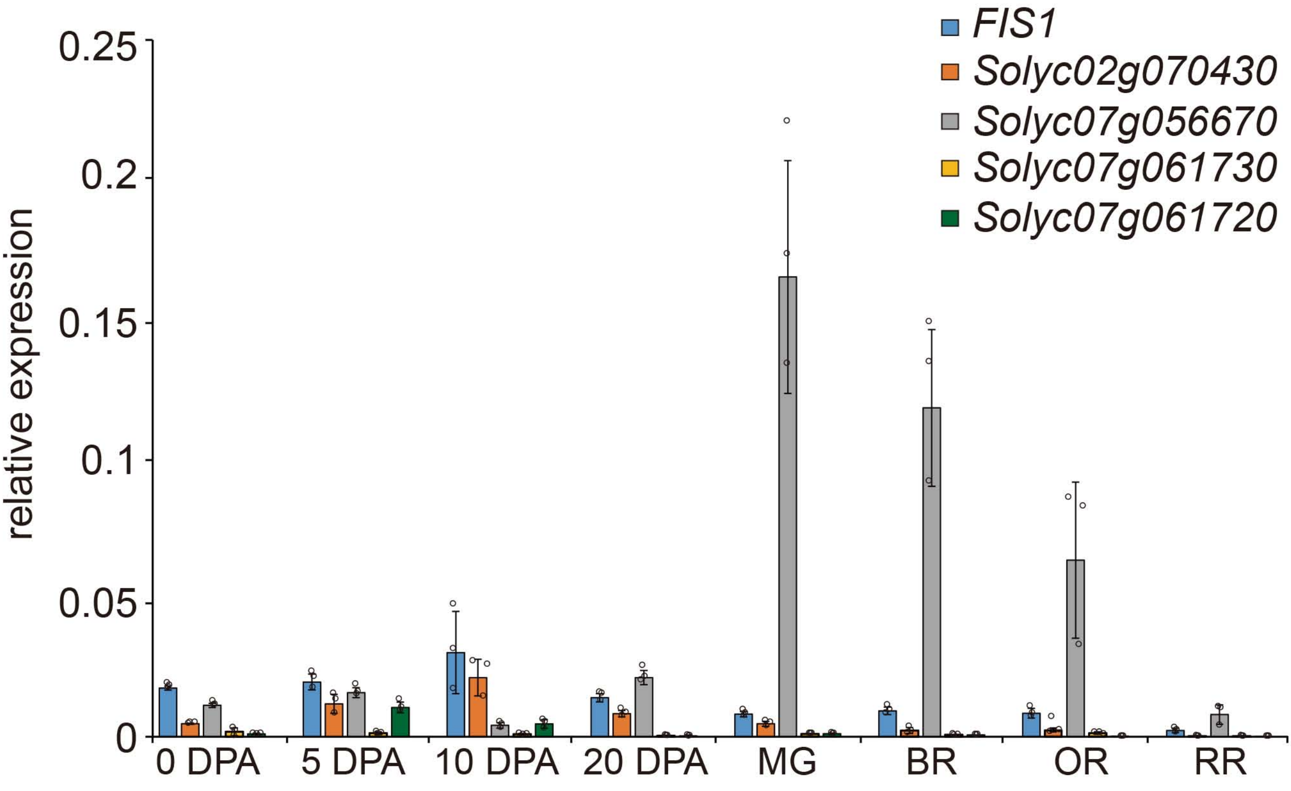

Supplementary Fig 10. Expression pattern of *FIS1* homolog genes in CC. Error bars, mean ± SD, n = three biological replicates. DPA, days post anthesis; MG, mature green; BR, breaker; OR, orange; RR, red ripe.

Supplementary Table 1. List of markers and primers used in the work.

Marker/ Primer ID	Gene name	Position/Gene ID (SL3.0)	Sequence (5'-3')	Application
M1	*FIS1*	ch10:1374787-1374956	CGATGAGCTCAACCCGATATGC<br>GATTCGATCCATCACGCTTTGG	Mapping
M2	*FIS1*	ch10:1813173-1813448	GTCGAGATACGTTGTTCCACCCTG<br>GTGACTGAGATTGGCATTACTTACCAG	Mapping
M3	*FIS1*	ch10:1840917-1841102	CGAAGGGATAGTATTACTCTCCATC<br>CACAAGAGTGATTTAGTGAGGCC	Mapping
M4	*FIS1*	ch10:2050957-2051253	CTAACAAGCTGTTTTGCCTTACTCTG<br>CTTGCTTTATCGGGATTTACCTGAAC	Mapping
M5	*FIS1*	ch10:2189044-2189329	GTTGAAATCGCGGCGACACGAAC<br>CGTGACTACTTGAATGCCACTTACAAT	Mapping
M6	*FIS1*	ch10:2370551-2370642	CCTTTCGTACAAACACAAACTAA<br>GGCAAAAAGGCTACTGCTTCATTG	Mapping
M7	*FIS1*	ch10:2756919-2757059	GATCAACAGATGTACTTGTCAAT<br>GAATGAATGCAATAAGTACCG	Mapping
M8	*FIS1*	ch10:1854643-1854789	CCTCCGTGCTCTTTCCTTACG<br>GCTATTTCATAGTAGGTTGTCGGAA	Mapping
M9	*FIS1*	ch10:1872338-1872455	CCTATTGTTTCGATTATTAATTTGTGG<br>CAAATTCTTAAATGCAATCAAGAACTA	Mapping
M10	*FIS1*	ch10:1876018-1876267	GTATCATCTATCACTGAAACTTG<br>CGATCGAAGCATAATAAGAAAC	Mapping
M11	*FIS1*	ch10:1949298-1949488	CGAAAGAAGTATTGAATTAATATCAGG<br>CGCACACACACAGATTTAATATCAG	Mapping
P1	*FIS1*	*Solyc10g007570*	ATATCACCTGCACACTTTGGCCCTTGACCCT<br>TTTGGCTAGTTTCAGAGCTATGCTGGAA<br>ATATCACCTGCACACTTTGGTAGGACCCCTT<br>GACCCTTTGTTTCAGAGCTATGCTGGAA	CRISPR/Cas9 genome-editing
P2	*FIS1*	*Solyc10g007570*	CAAGAATTACAAATCCCCTTTCCC<br>GTTACACCTCGACGGGTCCTAGTC	Identification of *fis1* mutants for seed1
P3	*FIS1*	*Solyc10g007570*	GGACCCCTTGACCCTTTTGGC<br>CCACTTAACGAGCTCTACTCGCATAC	Identification of *fis1* mutants for seed2
P4	*FIS1*	*Solyc10g007570*	ATGGTGGTGTTGTCTAGGCAAGTAGTTAA<br>CTTTAATTTCTCAAATAGGACCAACCTA	Promoter amplification
P5	*FIS1*	*Solyc10g007570*	TTGAGACAGCCCCACCTTACTCTTA<br>GGTCAAATTTTTTTGGAAATTAAAG	CDS amplification
P6	*FIS1*	*Solyc10g007570*	GCAGGTGATGACAAATGGGAGGTT<br>AAACGCATCTGCCAAATATTTTGC	Identification of complementary lines
P7	*FIS1*	*Solyc10g007570*	GGCCAATAGTGTAAAATCAAGACTC<br>CTTTGTACAAGCTGTCTTCATCCCC	Quantitative RT-PCR
P8	*CYP86A69*	Solc08g081220	TGAGCTGGTTCTTTTGGCTTG<br>TGTGTCGTTGCCACGTGTCT	Quantitative RT-PCR
P9	*CYP77A-like*	*Solyc03g119200*	GACAAAGTGGAAAACGTAGCTATTA	Quantitative RT-PCR

			ACGGCTCTGCTTCAAGAACTCAC	
P10	*GDSL-like*	*Solyc04g081770*	GTCAACTATAAGGTCATGTGAAGGG<br>ATCCCCAGTTCTTCAACTATCAAG	Quantitative RT-PCR
P11	*GDSL-like*	*Solyc03g111550*	ATGCATTATATCTATATAAACC<br>ATCAGTGTTATGGTGAATATGATG	Quantitative RT-PCR
P12	*CER1-like*	*Solyc01g088400*	CGAAGAAGACTACAAGAGACTTAATG<br>GTAGAAGCAATCCTTGCGAGA	Quantitative RT-PCR
P13	*CER26-like*	*Solyc07g006680*	CTCATTCTTCAATCCCACTCATT<br>TAAAATTGAGTTAAAGTTTGTG	Quantitative RT-PCR
P14	*CER4*	*Solyc11g067190*	ATCTTCTTTTAAGAGCTGCAGATGAC<br>TTGGTCCACATTTTTCTCTTAG	Quantitative RT-PCR
P15	*FIS1*	*Solyc10g007570*	GCCATAAAATTCTTTTCATCTCCA<br>CCCTCAGCTAACATTTCAAGAATC	Identification of *FIS1* gene structure
P16	*FIS1*	*Solyc10g007570*	GCATCTATTTTGGGTCTCAATCCA<br>CATCAGGACATGGAGGATAATG	*In situ* hybridization probe
P17	*FIS1*	*Solyc10g007570*	CACCATGGTGGTGTTGTCTAGGCAAGTAG<br>CTTTAATTTCTCAAATAGGACCAACC	Construction of enzyme activity vector

Supplementary Table 2. Mutation frequency analysis at predicted off-target sites of *FIS1*.

gRNA	Target sequence	PAM	Predicted off-target sequence	gene	locus	Off-target analysis
FIS1	GCCCTTGACCCTTTTGGCTA	TGG	CCTGCTGACCCTTTTGGCTATGG	*Solyc07g061730.3*	SL3.0ch07:+64824630	0/10
			CCTGCTAACCCTTTTGGCTATGG	*Solyc02g070430.3*	SL3.0ch02:-40726412	0/10
			CCATAGCCAAAAGGATCAGCAGG	*Solyc07g056670.3*	SL3.0ch07:-64603294	0/10
			TGGCTTGACCCTTTTGACTATGG	*Solyc03g116870.3*	SL3.0ch03:-67613281	0/10
			CCCCTTGCGCCTTTCGGCTTGGG	*Solyc07g054270.3*	SL3.0ch07:-62736216	0/10
			CCCCTTGACCAATTTGAATATGG	*Solyc02g021650.3*	SL3.0ch02:-23848650	0/10
FIS1	GATTAGACTAGGACCCGTCG	AGG	AATCAGACATGAACCCGTCGGGG	*Solyc05g008910.3*	SL3.0ch05:+3121105	0/10

Supplementary Table 3. The contents of cutin and wax compositions in fruit pericarps of NIL-*fis1*^{MM} and NIL-*FIS1*^{CC}.

		Mature green fruits		Red ripen fruits	
		**NIL-*FIS1*^{CC}**	**NIL-*fis1*^{MM}**	**NIL-*FIS1*^{CC}**	**NIL-*fis1*^{MM}**
**Cutin**	**Hexadecanoic**	6.77±0.69 ^{a}	7.74±1.86 ^{a}	8.84±0.72 ^{a}	7.00±0.67 ^{a}
	**p-courmaric**	2.76±0.18 ^{b}	2.92±0.10 ^{b}	3.58±0.17 ^{a}	3.84±0.12 ^{a}
	**Octadecanoic**	1.19±0.03 ^{a}	1.15±0.07 ^{a}	1.48±0.25 ^{a}	1.21±0.04 ^{a}
	**16-OH hexadecanoic**	34.95±1.52 ^{c}	67.09±2.78 ^{b}	60.19±7.75 ^{b}	93.01±2.88 ^{a}
	**9-OH pentadecanoic acid**	3.31±0.14 ^{a}	3.09±0.29 ^{a}	4.32±0.96 ^{a}	3.40±0.06 ^{a}
	**Hexadecane-1,16-dioic**	5.85±0.14 ^{c}	10.96±0.18 ^{b}	13.16±1.64 ^{b}	22.03±1.64 ^{a}
	**9,18-DiOH octadecanoic**	45.22±3.61 ^{a}	44.07±0.75 ^{a}	29.66±0.93 ^{b}	33.03±0.93 ^{b}
	**10,16-DiOH hexadecanoic**	760.94±70.28 ^{b}	1087.95±101.77 ^{a}	571.37±20.00 ^{c}	775.03±63.54 ^{b}
	**10-OH hexadecanoic**	62.95±2.16 ^{a}	77.95±0.34 ^{a}	53.36±0.74 ^{b}	70.10±1.53 ^{a}
	**9,10,18-TriOH octadecenoic***	13.22±0.42 ^{b}	15.75±0.43 ^{a}	7.52±1.01 ^{c}	15.36±0.12 ^{a}
	**9,10,18-TriOH octadecanoic**	30.21±0.72 ^{b}	37.10±2.26 ^{a}	26.36±3.71 ^{b}	35.73±0.74 ^{a}
	**Others**	8.3±1.18 ^{a}	7.57±1.25 ^{ab}	5.87±0.30 ^{c}	6.8±0.30 ^{b}
**Wax**	**Ester**	0.00±0.00 ^{b}	0.01± 0.00 ^{b}	0.03±0.01 ^{a}	0.03±0.02 ^{a}
	**FA**	0.38±0.24 ^{a}	0.23±0.17 ^{a}	0.67±0.54 ^{a}	0.18±0.10 ^{a}
	**Alcohol**	3.23±0.44 ^{b}	3.09±0.31 ^{b}	3.66±0.85 ^{b}	6.67±1.98 ^{a}
	**Alkane**	4.71±0.47 ^{c}	5.00±0.24 ^{c}	9.15±2.35 ^{b}	15.33±1.77 ^{a}
	**iso-Alkane**	0.29±0.06 ^{a}	0.30±0.07 ^{a}	0.26±0.15 ^{a}	0.33±0.09 ^{a}
	**Amyrin**	2.65±0.19 ^{b}	2.35±0.16 ^{b}	3.34±0.71 ^{ab}	5.06±1.49 ^{a}

Data represent the means values (μg/cm^2) are given with three replicates ± SD.

* , Double-bond position not determined.

Different letters indicate significant differences according to the Tukey-Kramer test ($P < 0.05$).

Supplementary Table 4. List of tomato accessions used to evaluate compression resistance.

Line number	Individual Code	Genotype	Origin
1	TS-031	CC	Honduras
2	TS-057	CC	Peru
3	TS-304	CC	Peru
4	TS-025	CC	Unknown
5	TS-129	CC	Peru
6	TS-034	CC	Peru
7	TS-148	CC	Peru
8	TS-040	CC	Unknown
9	TS-248	CC	Unknown
10	TS-028	CC	Mexico
11	TS-253	MM	United States
12	TS-203	MM	Italy
13	TS-236	MM	Russian Federation
14	TS-142	MM	Italy
15	TS-081	MM	Russia
16	TS-136	MM	Italy
17	TS-060	MM	United States
18	TS-174	MM	Unknown
19	TS-157	MM	Russia
20	TS-152	MM	Brazil
21	TS-204	MM	United States
22	TS-297	MM	China
23	TS-166	MM	Peru
24	TS-211	MM	United States
25	TS-218	MM	Brazil
26	TS-147	MM	Unknown
27	TS-043	MM	Unknown
28	TS-140	MM	Italy
29	TS-088	MM	Italy
30	TS-168	MM	Italy
