# Supplementary material for: FIS1 encodes a GA2-oxidase that regulates fruit firmness in tomato
Source: Nat Commun. 2020 Nov 17;11:5844. doi: 10.1038/s41467-020-19705-w (PMC7673020; doi:10.1038/s41467-020-19705-w)
Supplement: Supplementary file 2 — Descriptions of Additional Supplementary Files [file 41467_2020_19705_MOESM2_ESM.pdf]

Descriptions of Additional Supplementary Files

Supplementary data 1

Description: Differential expression genes in 30 DPA or breaker fruits between NIL-fis1MM and NIL-FIS1CC. FPKM, fragments per kilobase of transcript sequence per million base pairs sequenced. DPA, days post anthesis. BR, breaker. n = three biological replicates.
